# A Trivalent Live Vaccine Elicits Cross-Species Protection Against Acute Otitis Media in a Murine Model

**DOI:** 10.3390/vaccines12121432

**Published:** 2024-12-19

**Authors:** Haley Echlin, Amy Iverson, Abigail McKnight, Jason W. Rosch

**Affiliations:** Department of Host-Microbe Interactions, St. Jude Children’s Research Hospital, Memphis, TN 38105, USA; haley.echlin@stjude.org (H.E.); amy.iverson@stjude.org (A.I.); abigail.mcknight@stjude.org (A.M.)

**Keywords:** acute otitis media, live-attenuated vaccine, multiple otopathogens

## Abstract

**Background**: Acute otitis media (AOM) is a common pediatric infection worldwide and is the primary basis for pediatric primary care visits and antibiotic prescriptions in children. Current licensed vaccines have been incompletely ineffective at reducing the global burden of AOM, underscoring a major unmet medical need. The complex etiology of AOM presents additional challenges for vaccine development, as it can stem from multiple bacterial species including *Streptococcus pneumoniae*, *Haemophilus influenzae*, and *Moraxella catarrhalis*. As such, targeting multiple pathogens simultaneously may be required to significantly impact the overall disease burden. **Methods**: In this study, we aim to overcome this challenge by engineering a live-attenuated vaccine platform based on an attenuated mutant of *S. pneumoniae* that expresses *H. influenzae* and *M. catarrhalis* surface epitopes to induce protective immunity against all three pathogens. **Results**: The trivalent live-attenuated vaccine conferred significant protection against all three bacterial otopathogens as measured by seroconversion and the development of AOM, with the inclusion of the additional epitopes providing unexpected synergy and enhanced protection against *S. pneumoniae*. **Conclusions**: These data demonstrate a novel mechanism of introducing non-native immunogenic antigens into a live-attenuated vaccine platform to engender protection against AOM from multiple pathogenic species.

## 1. Introduction

Bacterial acute otitis media (AOM) is a frequent infection in children [1,2] causing over 700 million cases per year worldwide, with 75 percent of children experiencing at least one infection before the age of three. The leading bacterial species responsible for AOM are *Streptococcus pneumoniae*, *Haemophilus influenzae*, and *Moraxella catarrhalis* [3,4,5,6,7,8,9]. There has been notable success in developing vaccines targeting bacterial otopathogens, namely, with capsule-based conjugate vaccines greatly reducing the incidence of invasive disease by pneumococcus [10,11] and *H. influenzae* type B [12] in children and adults. While the pneumococcal conjugate vaccine has been very successful in preventing invasive disease, it has been less effective against otitis media [13] and does not decrease recurrent pneumococcal AOM [14]. The initial pneumococcal polysaccharide conjugate vaccine (PCV-7) effectively reduced the overall incidence of invasive disease [15,16] and partially reduced otitis media [17]. Expanding vaccine coverage from 7 to 13 serotypes (PCV-13) has further decreased pneumococcal AOM incidence, though significant disease burden persists [18]. To expand coverage beyond the pneumococcus, multiple vaccines have employed the conjugation of capsules from 10 or 11 pneumococcal serotypes to a surface-exposed lipoprotein of *H. influenzae* [19,20,21]. This strategy has somewhat decreased the incidence of AOM caused by both pneumococcus and *H. influenzae* [22,23,24,25]. Likewise, there has been considerable interest in investigating the potential utility of dual NTHi-Mcat vaccines that target unencapsulated *H. influenzae* (NTHi) and *M. catarrhalis* [26,27]. 

This phenomenon of reduced vaccine efficacy against AOM is not fully explained by serotype replacement with non-vaccine serotypes, as even vaccine serotypes continue to be isolated from pneumococcal AOM cases in vaccinated populations [19,28,29,30]. This issue may be due to poor mucosal antibody responses or the low expression of the capsular antigen during pneumococcal colonization [31]. The active removal of capsular polysaccharide in response to host antimicrobial peptides at the mucosal surface may also help *S. pneumoniae* evade anti-capsular antibodies conferred by vaccination [32]. Moreover, colonization with non-vaccine-serotype and non-typeable pneumococci and the predominance of NTHi have resulted in these pathogens continuing to be a significant medical burden for both community-acquired pneumonia [10,33] and AOM [34,35]. The recognition of diseases such as AOM with varying infectious etiologies underscores the value of exploring additional vaccine strategies that target several pathogens underlying the same disease to provide the broadest protective capacity.

The incidence of both mucosal and invasive pneumococcal disease decreases after early childhood, a phenomenon thought to be the result of accumulating protein antigen exposure leading to the development of broad protective antibody-mediated immunity [36]. There has been continued interest in the development of protein-based vaccines, which have demonstrated considerable promise both alone and in combination with capsule-based vaccines in conferring protective immunity against pneumococcal disease [37]. Another potential vaccine strategy that takes advantage of repeated exposure to bacterial antigens is the introduction of a live-attenuated vaccine in early childhood. Vaccination with live-attenuated strains acts as a means of exposure to multiple bacterial antigens, stimulating both humoral and cellular responses and, thereby, generating robust protection against both mucosal and invasive pneumococcal infection [38,39,40]. Many efforts have utilized highly attenuated vector strains to express important proteins and capsule-based pneumococcal antigens to induce protective immunity via either oral or intranasal administration. These have included approaches utilizing attenuated *Salmonella* vaccine platforms to express pneumococcal polysaccharides or protein antigens [41,42,43,44,45], commensal *Lactobacillus* and *Lactococcus* species to express pneumococcal antigens [46,47,48,49,50,51], vectors expressing either secreted or lipoprotein anchored pneumococcal antigens [52], and adenovirus vectors expressing multiple pneumococcal antigens [53]. These studies demonstrate that protective immunity against the pneumococcus can be elicited at the mucosal surface against subsequent disease development. In addition to the utilization of other bacterial species encoding pneumococcal antigens, there has been continued interest in modulating the pneumococcus to be avirulent whilst persisting at a sufficient density and duration to generate robust immunity and protection [38,39,40]. Initial studies using attenuated temperature-sensitive pneumococcal mutants demonstrated protection against challenge [54]. Subsequent investigations have utilized targeted deletions of the capsule locus coupled with mutations in iron or proline uptake systems, which induce robust antibody responses and confer serotype-independent immunity against invasive disease, though the deletion of the capsule appears to reduce heterologous protection during colonization [55,56]. Additional live-attenuated vaccines in which the capsule and DNA uptake machinery have been deleted induce both serum and mucosal antibody responses, with the additional benefit for the lack of reversion from recombination in a naturally competent pathogen [57]. Similar strategies have also proven successful via the modulation of virulence via the deletion of *pep27* while simultaneously inactivating competence [58]. Other approaches include vaccination with strains lacking prolipoprotein diacylglyceryl transferase, which results in heterologous protection against subsequent pneumococcal challenge [38,59]. However, this deletion results in the impaired trafficking of multiple pneumococcal lipoproteins, many of which have been explored as candidates for protein-based vaccines [60]. These studies underscore that pneumococcal attenuation presents considerable potential for advancing live vaccines to confer serotype-independent immunity against both invasive and mucosal disease.

Antibody responses alone are not always indicative of protective responses, as live vaccines that induce antibody responses against conserved protein antigens may not confer protection against subsequent heterologous challenge [61]. As such, additional considerations for using the pneumococcus as a vaccine platform are the colonization duration of the vaccine strain, as colonization duration is a key aspect in generating protective immunity [62], and the retention of the major immunogenic surface proteins, many of which are virulence factors whose deletion may impact vaccine. The deletion of key virulence factors in certain serotypes have been shown to confer effective protective capacity while maintaining colonization [40]. Other strategies addressing these concerns include reducing virulence via synthetic changes in virulence factor codon-pair bias to reduce pathogenicity while retaining the native protein sequence [63]. In this study, we describe the advancement in a pneumococcal live vaccine platform based upon the deletion of *ftsY*, a key component of the Signal Recognition Particle Pathway that was previously demonstrated to be highly effective in conferring protective immunity against both invasive disease and AOM in a serotype-independent manner [39]. The vaccine strain was highly attenuated for invasive disease, while maintaining nasal carriage, and was found to be recalcitrant to genetic reversion, due to a pronounced defect in natural competence. Underscoring the importance of *ftsY* in pneumococcal fitness, horizontal transfer of the deletion mutation into other clinical strains was highly restricted, with many strains demonstrating markedly reduced or inability to uptake of the mutation. Prior colonization or administration of a conjugate vaccine did not reduce the vaccine efficacy. Moreover, as AOM is a disease caused by multiple bacterial pathogens, we engineered the pneumococcal *ftsY* mutant strain to express epitopes from both *H. influenzae* and *M. catarrhalis* on the bacterial surface, resulting in increased vaccine efficacy against the pneumococcus and protection against *H. influenzae* and *M. catarrhalis*. These data suggest that multiple bacterial pathogens of the mucosa can be targeted via the expression of the respective antigens in a live-attenuated pneumococcal vaccine platform to confer cross-species protection against AOM. 

## 2. Materials and Methods

### 2.1. Bacterial Cultivation

*Streptococcus pneumoniae* strains were grown on solid TSA (Tryptic soy agar; Millipore Sigma, Burlington, MA, USA) supplemented with 3% sheep blood (Lampire Biologicals, Everett, PA, USA) and 20 μg/mL neomycin at 37 °C in a 5% CO_2_ atmosphere. Overnight growth was directly inoculated into semi-synthetic casein liquid media and 0.5% yeast extract (C + Y) [64] and grown in static conditions at 37 °C in a 5% CO_2_ atmosphere. Non-typeable *Haemophilus influenzae* 86-028NP was grown on solid chocolate agar media supplemented with 11,000 units/L bacitracin (Remel, San Diego, CA, USA) at 37 °C in a 5% CO_2_ atmosphere. Overnight growth was directly inoculated into brain–heart infusion broth (BD) supplemented with 0.2% yeast extract (BD) (BHI + Y), 10 μg/mL NAD+ (Sigma, St. Louis, MO, USA), and 10 μg/mL hemin (Sigma) with aeration. *Moraxella catarrhalis* strain O35E was grown in BHI + Y overnight at 37 °C with aeration. The strains used in this study are listed in Table S1. To determine growth kinetics, frozen stocks were diluted 1:100 in C + Y in a 96-well plate, and OD_620_ was measured every 30 min for 30 h using a Biotek Cytation 3 plate reader. The growth curves of the LAV strains were compared to those of BHN97 via two-way ANOVA, repeated measures using Prism 10 (GraphPad, San Diego, CA, USA).

### 2.2. Genetic Constructs

Capsule-swapped strains were generated as previously described [65]. Briefly, the TIGR4::19F capsule swap variant was generated through the transformation of an unencapsulated TIGR4SΔcps::NewSweetJanus with genomic DNA (gDNA) from BHN97 (19F). To obtain the capsule-swapped variants in the BHN97 background, an unencapsulated BHN97Δcps::SweetJanus strain [66] was transformed with either BHN97 (19F) or TIGR4 (4) gDNA.

For the generation of vaccine strains, all PCR products were amplified using exTaq polymerase (TAKARA) following the recommended guidelines; the primers are listed in Table S2. gDNA was extracted using the aqueous/organic extraction protocol as described previously [65]. For all transformations, *S. pneumoniae* strains were grown in C + Y until OD620 = 0.08 and were incubated with CSP1 and CSP2 [67] and the specific amplicon for three hours at 37 °C in a 5% CO_2_ atmosphere. The entire culture was plated on selection plates, and the correct transformants were confirmed through a lack of growth on counter-selection plates and through PCR. For transformations including the PhunSweetErm cassette, the selection plates contained 1 µg/mL erythromycin, and the counter-selection plates contained 15mM chlorinated-phenylalanine and 10% sucrose. For transformations replacing PhunSweetErm, the selection plates contained 15mM chlorinated-phenylalanine and 10% sucrose, and the counter-selection plates contained 1 µg/mL erythromycin as previously described [65,68].

To generate the live-attenuated *S. pneumoniae* strain (LAV), an internal deletion of *ftsY* (Sp1244) was generated, following a similar strategy employed previously [39]. The internal 867 nucleotides of *ftsY* were replaced with PhunSweetErm [68] through splicing-by-overlap-extension (SOE) PCR [69]. The upstream flanking region of *ftsY* and the first 213 bp of *ftsY* were amplified from BHN97 gDNA using the primer pair FtsY_Up_F/FtsY_Up_R. The downstream flanking region of *ftsY* and the last 210 bp of *ftsY* were amplified from BHN97 gDNA using the primer pair FtsY_Down_F/Fsy_Down_R. The Δ*ftsY*::PhunSweetErm amplicon was generated through SOE PCR using the upstream, downstream, and PhunSweetErm fragments as the template and the primer pair FtsY_Up_F/FtsY_Down_R. BHN97 was transformed with the Δ*ftsY*::PhunSweetErm amplicon to obtain the LAV strain.

To generate the LAV-D strain, BHN97 underwent three transformations steps. For the first transformation, an expression platform in the neutral CEP locus [70,71] was generated through the insertion of PhunSweetErm. The flanking regions in the CEP were amplified using BHN97 gDNA and the primer pair CEP_Up_F/CEP_Up_PS_R as well as CEP_Down_PS_F and CEP_Down_R. Of note, these regions are shared among several *S. pneumoniae* strains; however, in BHN97, an insertion at this site disrupts predicted ATP transporter proteins. The CEPΩPhunSweetErm amplicon was generated through SOE PCR using the CEP upstream, CEP downstream, and PhunSweetErm amplicons as the template and the primer pair CEP_Up_F/CEP_Down_R and was used to transform BHN97 to generate BHN97 CEPΩPhunSweetErm. For the second transformation step of generating the LAV-D strain, a codon-optimized gene expressing *H. influenzae* ProteinD (*glpQ* in non-typeable 86-028NP) [72] was inserted in the CEP locus, replacing PhunSweetErm, thereby making the strain markerless. Three strategies were employed to anchor ProteinD to the cell surface, i.e., with C-terminal LPXTG motif (ProteinD-LPXTG), with C-terminal choline-binding motif (ProteinD-CBD), and with N-terminal lipoprotein anchor domain (Lipo-ProteinD). For ProteinD-LPXTG and ProteinD-CBD, a Gram-positive signal sequence was incorporated into the N-terminus. All three *proteinD* genes were synthesized via Genscript (Piscataway) with codon optimization for *S. pneumoniae* and included a strong promoter (P3) and upstream and downstream terminators described previously [71] (see Figure S3 for protein sequences). The flanking regions in the CEP were amplified using BHN97 gDNA and the primer pairs CEP_Up_F/CEP_Up_D_R and CEP_Down_D_F/CEP_Down_R. The codon-optimized *proteinD* with the CBD and LPXTG motifs, along with the P3 promoter and terminators, were amplified from Genscript plasmids using the primer pair ProteinD_F/ProteinD_R. For the *lipo-proteinD* amplicon, Genscript was unable to synthesize the entire sequence and instead provided two fragments in two separate plasmids. To generate the entire sequence of *lipo-proteinD*, along with the P3 promoter and terminators, the two fragments were amplified from the plasmids using the primer pairs Lipo-ProteinD_1_F/Lipo-ProteinD_1_R and Lipo-ProteinD_2_F/Lipo-Protein_2_R. The two fragments were spliced together via SOE PCR using the primer pair Lipo-ProteinD_1_F/Lipo-ProteinD_2_R. The CEPΩ*proteinD-LPXTG*, CEPΩ*proteinD-CBD*, and CEPΩ*lipo-proteinD* amplicons were generated through SOE PCR using the CEP upstream, CEP downstream, and *proteinD-LPXTG*/*proteinD-CBD*/*lipo-proteinD* amplicons as the template and the primer pair CEP_Up_F/CEP_Down_R. To generate BHN97 strains expressing differentially anchored ProteinD, BHN97 CEPΩPhunSweetErm was transformed using the CEPΩ*proteinD-LPXTG*, CEPΩ*proteinD-CBD*, or CEPΩ*lipo-proteinD* amplicons. For the third transformation step, the internal 867 nucleotides of *ftsY* were replaced with PhunSweetErm. The Δ*ftsY*::PhunSweetErm amplicon was amplified from BHN97Δ*ftsY*::PhunSweetErm gDNA using the primer pair FtsY_Up_F/FtsY_Down_R. BHN97 CEPΩ*lipo-proteinD* was transformed with the Δ*ftsY*::PhunSweetErm amplicon to obtain the LAV-D strain.

To generate the LAV-D-M strain, the BHN97 CEPΩPhunSweetErm generated above underwent two transformation steps. For the first step, a gene expressing Lipo-ProteinD with an additional 23 amino acid UspA epitope (“NNINNIY”) [73] on the C-terminus was generated using SOE PCR. The upstream fragment was amplified from the gDNA of BHN97 CEPΩ*lipo-proteinD* using the primers CEP_Up_F and Lipo-ProteinD_N_R such that the amplicon contained the upstream CEP flanking region and the *lipo-proteinD* gene. A 23 double-stranded ultramer fragment of the NNINNIY epitope was generated by annealing two ultramer oligos together, NNINNIY_Ultra_F and NNINNIY_Ultra_R. The downstream fragment was amplified from the gDNA of BHN97 CEPΩ*lipo-proteinD* using the primers CEP_Down_N_F and CEP_Down_R such that the amplicon contained the terminators downstream of the *lipo-proteinD* gene and the downstream CEP flanking region. To enhance overlap during SOE PCR of the final product, the upstream and NNINNIY ultramer fragment were spliced together using the primers CEP_UP_F and NNINNIY_R, and the downstream and NNINNIY ultramer fragment were spliced together using the primers NNINNIY_F and CEP_Down_R. The final product, the CEPΩ*lipo-proteinD-M* amplicon, was generated through SOE PCR using the CEP upstream-NNINNIY and the CEP downstream-NNINNIY amplicons as the template and the primer pair CEP_Up_F/CEP_Down_R. BHN97 CEPΩPhunSweetErm was transformed using the CEPΩ *lipo-proteinD-M* amplicon. For the next transformation step, the internal 867 nucleotides of *ftsY* were replaced with PhunSweetErm using the same method as that for the LAV-D strain. BHN97 CEPΩ*lipo-proteinD-M* was transformed with the Δ*ftsY*::PhunSweetErm amplicon to obtain the LAV-D-M strain.

### 2.3. Recombinant ProteinD Purification and Antibody Generation

To purify ProteinD for antibody generation, an *E. coli* codon-optimized *proteinD* was synthesized with an N-terminal His-tag in a pet28 expression plasmid by Genscript (see Table S3 for protein sequence). The pet28-*his-proteinD* plasmid was transformed into One Shot BL21 (DE3) chemically competent *E. coli* cells following manufacturer’s recommended protocol (Thermo Fisher, Waltham, MA, USA). Cells harboring pet28-*his-proteinD* were grown in 2TXY (2%Tryptone, 1% yeast extract, and 0.5% NaCl) media and 30 µg/mL kanamycin, and when the cells reached OD600 = 0.6, their protein expression was induced with 0.2 mM IPTG for 16 h at 18 °C. The culture was pelleted and lysed in lysis buffer (1x PBS, pH 7.4, and protease inhibitors) via a microfluidizer. The lysed pellet were incubated with nickel beads and washed, and His-ProteinD was eluted from beads using 200 mM imidazole in PBS. Rabbit polyclonal antibody against ProteinD was generated by Rockland Immunochemicals. Briefly, rabbits were immunized with 400 µg of recombinant His-ProteinD, followed by three boost vaccinations of 200 µg of recombinant His-ProteinD at two, three, and five weeks post initial vaccination. The rabbits were bled 12 weeks post final vaccination for the collection of sera.

### 2.4. Recombination Frequency

To determine the competence of the LAV strain, BHN97 and the LAV strain were transformed with 5 µg of gDNA from BHN97 Tn-seq, a nontargeted mutagenesis mechanism containing a spectinomycin resistance cassette [74]. Strains were grown in C + Y until OD620 = 0.08, and this was followed by transformation as described above. The transformations were serially diluted and plated on TSA blood plates and TSA blood plates supplemented with 200 µg/mL spectinomycin. To observe low-frequency recombination events, the transformation was also spread-plated on TSA blood plates supplemented with 200 µg/mL spectinomycin. The experiment was repeated eight times. To measure potential spread of the antibiotic resistance cassette originating from the LAV strain, a subset of *S. pneumoniae* strains representing different serotypes was transformed with either 3 µg of gDNA from the LAV strain or with 3 µg of gDNA from BHN97 CEPΩPhunSweetErm, which served as a neutral control for the antibiotic resistance cassette. The cells were incubated with either CSP1 (D39), CSP2 (TIGR4), or both (all other strains) and the gDNA for 3 h at 37 °C, 5% CO_2_, followed by serial dilution and plating on TSA blood plates and TSA blood plates supplemented with 1 µg/mL erythromycin. To observe low-frequency recombination events, the transformation was also spread-plated on TSA blood plates supplemented with 1 µg/mL erythromycin. The experiment was repeated six times. The plates were incubated overnight at 37 °C in a 5% CO_2_ atmosphere, and colonies were enumerated. Recombination frequency was calculated as the number of recombinants on the plates containing the selection antibiotic divided by the total number of cells on the plates without the selection antibiotic and was compared via non-parametric Mann–Whitney *t*-tests using Prism 10 (GraphPad).

### 2.5. Adhesion Assay

Pneumococcal adherence to A549 lung epithelial cells was determined from a modified adhesion assay [75]. A549 cells (ATCC) were seeded to ~95% confluency (2 × 10^5^ cells/mL) in 24-well tissue-culture-treated plates (Costar). The cells were treated for 2 h prior to infection with TNF-α (10 ng/mL) in F12K media (ATCC) with 10% FBS and 20 µg/mL gentamicin, followed by a 2x rinse with PBS. *S. pneumoniae* strains were grown in C + Y until OD620 = 0.4 and diluted 1:10 into F12K. The A549 cells were infected with the diluted bacterial culture at an MOI of ~50:1. The bacteria were allowed to adhere to the A549 cells for 30 or 90 min at 37 °C. Nonadherent bacteria were determined by serial dilution of the supernatant and subsequent plating on TSA blood agar plates. The A549 cells with adherent bacteria were washed with PBS and detached from the wells via incubation with 100 µL of 0.1% Trypsin in PBS at 37 °C for 5 min, followed by the addition of 900 µL of PBS. The adherent bacteria were determined by serial dilution of the cell solution in PBS and subsequent plating on TSA blood agar plates. Each strain was measured in five biological replicates per plate, in two independent experiments. Adherence was reported as the number of adherent bacteria divided by the total number of bacterial cells (nonadherent and adherent). The number of adherent colonies/total bacterial cells of the LAV strains was compared with that of wild-type BHN97 via non-parametric Mann–Whitney *t*-tests using Prism 10 (GraphPad).

### 2.6. Vaccination Regimens and Protection Efficacy

For all vaccinations, treatments, and challenges via intranasal (IN) installation, mice were anesthetized under inhaled 2.5% isoflurane. For the vaccination of mice with the LAV strains, bacterial strains were grown in C + Y until OD_620_ = 0.4. Bacterial cultures were pelleted and diluted in PBS to 10^5^ CFUs/25 µL according to a previously determined standard curve. The bacteria were enumerated on TSA blood agar plates to confirm the inoculum. Eight-week-old female BALB/c mice (Jackson laboratory, Bar Harbor, ME, USA) were administered the vaccine via IN instillation of 10^5^ CFUs in 25 µL of PBS of the LAV strains or 25 µL of PBS as a control [39]. Three weeks and six weeks after the initial vaccination, the mice were boosted with a second and third IN instillation of 10^5^ CFUs in 25 µL of PBS of the LAV strains or 25 µL of PBS as above. For the co-vaccination of mice with Prevnar-13, 8-week-old female BALB/c mice were administered 100 µL of a 1:10 dilution of Prevnar-13 via intraperitoneal injection while conscious. Three, six, and nine weeks following Prevnar-13 vaccination, the mice were vaccinated with the LAV strains following the regimen above. As a control, a separate group of mice were administered 100 µL of a 1:10 dilution of Prevnar-13 via intraperitoneal injection followed by IN instillation of PBS at 3, 6, and 9 weeks. To determine the impact of previous colonization on vaccination efficacy, the bacterial strains were grown in C + Y until OD_620_ = 0.4. These strains included a homologous strain (BHN97) expressing its own capsule (Type 19F) as well as a variant capsule (Type 4) and a heterologous strain (TIGR4) expressing its own capsule (Type 4) as well as a variant capsule (Type 19F). Eight-week-old female BALB/c mice (Jackson laboratory) were administered the colonizing strains via IN instillation of 10^4^ CFUs in 25 µL of PBS or 25 µL of PBS as a control. Three weeks following colonization, the mice were vaccinated with the LAV strains following the regimen above. The mice were monitored for indications of distress or disease over the course of the vaccination regimen, and no observable signs were detected. Prior to the initial vaccination and two weeks after the final vaccination, sera were collected via non-lethal retro-orbital bleeding to measure seroconversion. Three weeks after the final vaccination, the mice were challenged. For all challenge experiments, the mice were administered the challenge strain via IN instillation. The vaccinated mice were challenged with 10^6^ CFUs/100 µL PBS of homologous *S. pneumoniae* strain (BHN97x) or heterologous *S. pneumoniae* strain (BHN54x) via IN instillation. For vaccination with LAV-D, an independent group of mice were challenged with *H. influenzae*, and for vaccination with LAV-D-M, independent groups of mice were challenged with either *H. influenzae* or *M. catarrhalis*. Similar to previous studies that leveraged influenza co-infection to facilitate NTHi translocation into the middle ear [76], we utilized an inflammatory stimulant poly (I:C) [77] to facilitate translocation to the middle ear in our challenge model as we did not observe appreciable middle ear translocation for either *H. influenzae* or *M. catarrhalis* without prior poly (I:C) sensitization (Figure S5). The mice were administered 50 μL of 1 mg/mL poly(I:C) HMW (Invivogen) IN daily for four days [68] followed by IN challenge with 10^7^ CFUs/100 µL PBS of *H. influenzae* or *M. catarrhalis* grown in liquid media. Twenty-four hours after all the challenges, the mice were euthanized via CO_2_ asphyxiation followed by cervical dislocation. The lungs, nasal passages, and both ear bullae were immediately harvested, homogenized in PBS, serially diluted, and plated on TSA blood agar plates and 400 µg/mL kanamycin plates (*S. pneumoniae*), chocolate agar plates (*H. influenzae*), or BHI agar plates (*M. catarrhalis*). Plates were incubated overnight at 37 °C in a 5% CO_2_ atmosphere, and the bacterial titers were enumerated. The bacterial burden in each tissue (CFUs/mL) was compared using a non-parametric Mann–Whitney *t*-test using Prism 10 (GraphPad). The bacterial burden in each ear was determined and plotted individually [78].

### 2.7. Murine Challenge Experiments 

To determine the virulence of the LAV strains in vivo, 8-week-old female BALB/c mice were infected via IN instillation of 10^5^ CFUs/25 µL PBS of the wild-type BHN97 or LAV strains (N = 10). At 3 days post-challenge, the mice were euthanized via CO_2_ asphyxiation followed by cervical dislocation, and the lungs, nasal passages, and both ear bullae were immediately harvested and homogenized in PBS. The lungs and ears were serially diluted and plated on TSA blood agar plates. The nasal passages were serially diluted and plated, and the remainder was spread-plated on TSA blood agar plates. The plates were incubated overnight at 37 °C in a 5% CO2 atmosphere, and the bacterial titers were enumerated. The bacterial burden in each tissue (CFUs/mL) was compared using a non-parametric Mann–Whitney *t*-test using Prism 10 (GraphPad).

To investigate the potential reversion of the LAV strain in vivo, the bacterial outgrowth from the spread-plated nasal passages above was considered Passage 1 (P1) in an in vivo passaging experiment. The bacterial growth on the blood agar plates was collected in C + Y, mixed with glycerol at a final concentration of 20%, and frozen at −80 °C in several aliquots. A frozen aliquot of each P1 sample served as the inoculum to challenge the next group of mice for the subsequent passage. The six highest titered stocks of each group were used for infection of 8-week-old female BALB/c mice via IN instillation of 10^5^ CFUs/25 µL PBS, N = 1 per P1 sample. At 3 days post-challenge, the mice were euthanized, and the lungs, nasal passages, and both ear bullae were harvested and processed as above. The bacterial burden was designated as Passage 2 (P2). Again, the bacterial outgrowth from the nasal passage was collected, frozen at −80 °C, and used to infect 8-week-old female BALB/c mice via IN instillation of 10^5^ CFUs/25 µL PBS, N = 1 per P2 sample. At 3 days post-challenge, the mice were euthanized and the lungs, nasal passages, and both ear bullae were harvested and processed as above. The bacterial burden was designated as Passage 3 (P3). For each strain and tissue, the bacterial burden of the sequential passages was compared using Kruskal–Wallis one-way ANOVA using Prism 10 (GraphPad). For all collected passages, a frozen aliquot was used to inoculate C + Y media and grown to OD620 = 0.6. gDNA was extracted, and the deletion of *ftsY* was confirmed via PCR using primer pairs FtsY_Outer_F/FtsY_Outer_R and FtsY_Inner_F/FtsY_Inner_R.

### 2.8. Western Blots

Bacterial cultures were grown in C + Y until OD620 = 0.6. The culture was pelleted, and the pellet was lysed in 10% deoxycholate and 10% sodium dodecyl sulfate at 37 °C to disrupt the cellular membranes. The bacterial lysates were diluted in PBS, boiled with 4x LDS sample buffer (Thermo Fisher), and run on 4–12% NuPAGE Bis-Tris gels (Invitrogen, Waltham, MA, USA). Proteins were transferred to nitrocellulose membranes, which were subsequently blocked with 5% non-fat skim milk in PBS + 0.1% Tween. To determine ProteinD expression in *S. pneumoniae*, the membranes were probed with anti-ProteinD polyclonal antibody, purified through ProteinA columns (Pierce, Franklin, MA, USA), at a concentration of 3 µg/mL in blocking buffer. As a loading control, the samples were concurrently run in a separate gel, transferred, and the membrane was probed with anti-CbpA 3H11 monoclonal [79] in blocking buffer. The membranes were incubated with all primary antibodies overnight at 4 °C and then probed with anti-mouse (for monoclonal primary antibody) or anti-rabbit (for polyclonal primary antibody) HRP-conjugated secondary antibodies (Bio-Rad, Hercules, CA, USA), followed by detection using an HRP chemiluminescent substrate (Thermo Scientific, Waltham, MA, USA).

### 2.9. ELISA Antibody Measurements

To determine ProteinD expression in *S. pneumoniae*, strains were grown in liquid media until OD_620_ = 0.4. The cells were pelleted, resuspended in equal volumes of coating buffer (sodium carbonate–bicarbonate; Sigma), and bound to high-binding Nunc 96-well plates (Thermo Fisher) via centrifugation. The supernatant was removed, and the plates were allowed to dry at room temperature and were subjected to blocking buffer (10% heat-inactivated fetal bovine serum in PBS). After blocking, the cells were probed with anti-ProteinD polyclonal antibody (purified through Pierce IgG ProteinA columns). As a control for differential plate-binding in the strains, cells bound to other wells in the same plate were concurrently probed with polyclonal antibody against LytA, a cell surface antigen [32]. The samples in the wells were washed with Tris-buffered saline and 1% Tween-20 and then probed with AP-conjugated goat anti-rabbit IgG secondary antibody (Southern Biotech, Birmingham, AL, USA) in blocking buffer. The samples in wells were washed again and incubated with AP yellow substrate (Sigma), and this was followed by detection at OD_405_ with a Biotek Cytation 3 plate reader. The absorbance values of the cells probed with ProteinD were normalized to those of the cells probed with LytA. The immunoreactivity, as detected by absorbance at 405 nm, of ProteinD-expressing strains and LAV strains were compared to that of BHN97 via an unpaired *t*-test using Prism 10 (GraphPad).

The immunoreactivity of mouse sera post-vaccination was determined via whole-cell bacterial and Protein-D ELISA. Strains were prepared and bound to 96-well plates as above. Sera from the vaccinated mice were diluted 1:50 and incubated with the bound bacteria. The samples in the wells were washed, probed with AP-conjugated goat anti-mouse IgG secondary antibody (Southern Biotech) in blocking buffer, and incubated with AP yellow substrate (Sigma), followed by detection at OD_405_ with a Biotek Cytation 3 plate reader. The immunoreactivity of the LAV strains was compared with that of BHN97 with an unpaired *t*-test using Prism 10 (GraphPad).

## 3. Results

### 3.1. Genetic Stability of LAV

**Figure 1:**
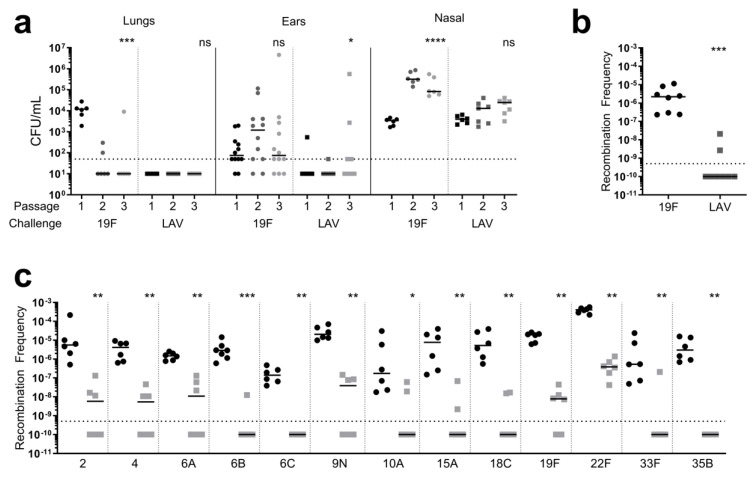
LAV is recalcitrant to reversion and is not readily obtained by other pneumococcal serotypes. (**a**) 19F (circles) and LAV (squares) strains were passaged in vivo via murine nasal colonization. Six mice were intranasally infected, and the lungs, ears, and nasal passages were harvested 3 days post-challenge. The bacterial burden was determined, and the bacterial growth on plates from the nasal passages were recovered (Passage 1). The recovered bacterial population from the nasal passage was used to challenge the mice for the next passage (2); N = 1 per each Passage 1 population. The bacterial population was passaged again using the same method (Passage 3). Each data point represents an individual mouse (lungs and nasal passage) or each ear from individual mice (ear), and the bars represent the median. The dashed line represents the limit of detection. For each strain and tissue, the bacterial burden of the sequential passages was compared via Kruskal–Wallis one-way ANOVA. (**b**) The competence of the LAV strain was determined by calculating the recombination frequency upon transformation with nontargeted Tn-seq gDNA. Each data point represents an individual biological replicate, and the bars represent the median. The dashed line represents the limit of detection. The recombination frequencies of the wild-type 19F and the LAV strain were compared via a non-parametric Mann–Whitney *t*-test. (**c**) The frequency of resistance spread from the LAV strain was determined by transforming strains of different serotypes with the LAV strain’s gDNA (squares, right). As a control, the same strains were transformed with gDNA from a strain harboring the resistance cassette at a neutral location (circles, left). Each data point represents an individual biological replicate, and the bars represent the median. The dashed line represents the limit of detection. For all strains, the recombination frequency of the strains transformed with the gDNA of the LAV strain was compared to that of the strains transformed with the control DNA via a non-parametric Mann–Whitney *t*-test. * *p* < 0.05, ** *p* < 0.01, *** *p* < 0.001, **** *p* < 0.0001, ns = non-significant.

The live-attenuated *ftsY* mutant in *S. pneumoniae* 19F (LAV) induces protection against pneumococcal infection when challenged with both a homologous and heterologous serotype, demonstrated through both seroconversion and reduced bacterial burden in the vaccinated mice (Figure S1), recapitulating observations made previously [39]. A key potential shortcoming of a live-attenuated pneumococcal vaccine is the inherent capacity of the pneumococcus to uptake and combine foreign DNA [80]. This presents a potential risk for reversion of the LAV strain to wild-type virulence. To address this concern, we first monitored the fitness of the LAV strain during repeated murine colonization (Figure 1). The vaccine strain remained attenuated compared to wild-type 19F (Passage 1) in the lungs and ears between the first and last passage, while maintaining bacterial burden levels in the nasal passage (Figure 1a). The passaged LAV bacterial populations were confirmed to maintain the *ftsY* deletion through PCR. Interestingly, the wild-type 19F became attenuated in the lungs between the first and final passage, as observed previously whereby adaptation to murine nasal colonization confers fitness tradeoffs for lung infection [81]. These data suggest that the LAV strain remains attenuated for invasive disease and AOM despite repeated passages in vivo. 

Next, we assessed the possibility of reversion of the LAV strain through a recombination event whereby the wild-type allele of a co-colonizing strain could replace the *ftsY* deletion. The level of competence in the LAV strain was determined by measuring the recombination frequency using a saturated Tn-seq library. The LAV strain demonstrated a severe defect in the recombination frequency of approximately four logs compared to the wild-type strain (Figure 1b). While the recombination of the saturated Tn-seq library in the LAV strain still occurred, genetic reversion due to recombination would not likely occur in the host considering the greatly reduced rate in ideal in vitro conditions, the growth defect imposed by the loss of *ftsY* (Figure S2a), and the lack of observable revertants when the LAV strain was passaged in the mice (Figure 1). Due to this pronounced reduction in competence, genetic manipulations for removing the erythromycin cassette were not successful, despite numerous attempts. This presents another potential risk of utilizing this strain as a live-attenuated vaccine in the unintentional spread of the resistance marker used in strain construction to non-vaccine strains during co-colonization. To determine the likelihood of resistance transfer, the frequency at which a panel of distinct serotypes recombined the antibiotic resistance marker from the gDNA of the LAV strain was measured (Figure 1c). For all serotypes, the frequency of recombining the resistance marker from the LAV strain was below levels of detection or severely impaired compared to obtaining the same resistance from a strain harboring the resistance marker in a neutral location. Of note, for three of the serotypes (2, 10 N, and 22 F), additional colonies were observed after 48 h of growth, demonstrating severe growth impairment upon obtaining the resistance from the gDNA of the LAV strain. These data underscore the genetic stability of the LAV strain and the inherently low risk of resistance mechanisms spreading to co-colonizing pneumococci.

### 3.2. Impact of Prior Colonization on LAV Efficacy

**Figure 2:**
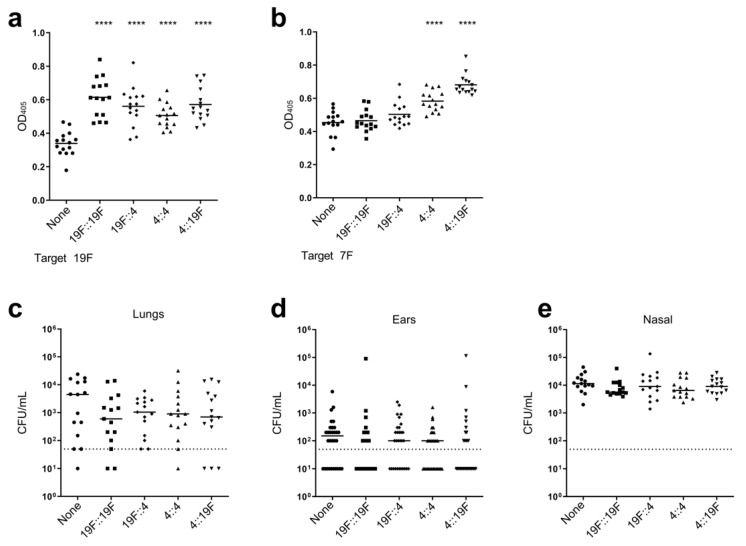
Prior colonization in mice is not detrimental to the protective efficacy of the LAV strain. (**a**–**e**) Mice (N = 15) were colonized with either a homologous strain (19F) expressing its own capsule (Type 19F; squares) or a variant capsule (Type 4; circles), or a heterologous strain (4) expressing its own capsule (Type 4; up triangles) or a variant capsule (Type 19F; down triangles). As a control, mice received a PBS vehicle and were not colonized (none; circles). Three weeks later, all the mice were vaccinated with the LAV strain. Following vaccination, all the mice were challenged with a 19F strain. (**a**,**b**) Sera were collected prior to challenge, and IgG seroconversion in the vaccinated mice was determined by ELISA against 19F (**a**) or 7F (**b**). Each data point represents an individual mouse, and the bars represent the mean. The immunoreactivity of sera from the mice colonized with each strain was compared to that of the non-colonized mice via an unpaired *t*-test. (**c**–**e**) Post-challenge with 19F, the bacterial burden in the lungs (**c**), ears (**d**), and nasal passage (**e**) of the vaccinated mice was determined. Each data point represents an individual mouse (**c**,**e**) or each ear from individual mice (**d**), and the bars represent the median. The dashed line represents the limit of detection. The bacterial burden in each tissue from the mice colonized with each strain was compared to that of the non-colonized mice via a non-parametric Mann–Whitney *t*-test. **** *p* < 0.0001. No significant difference was observed for any comparison without a designated *p* value.

Colonization by *S. pneumoniae* occurs early in childhood [82,83]. Taking this into consideration, it is imperative that the impact of prior colonization on the efficacy of the LAV strain be determined. To model this, mice were pre-colonized with pneumococcal strains followed by vaccination with the LAV strain. The impact of several pre-colonization strains on vaccine efficacy was determined, including a homologous strain (serotype 19F) and a heterologous strain (serotype 4). As *S. pneumoniae* has the inherent ability to swap capsule loci and express a distinct serotype [84], we also determined the impact of pre-colonization with capsule-swapped variants on vaccine efficacy (Figure 2). Serum antibody titers following vaccination with the LAV strain demonstrated that the pre-colonized and vaccinated mice had either similar or enhanced IgG levels against homologous (Figure 2a) and heterologous serotypes (Figure 2b) compared to the non-colonized and vaccinated mice. Following challenge, we observed a slight enhancement in protection against bacterial burden in the lungs and ears of the pre-colonized, vaccinated mice compared to the non-colonized, vaccinated mice (Figure 2c–e). These data suggest that prior colonization is not detrimental to the protective efficacy of the LAV strain and may enhance the protective capacity.

### 3.3. Impact of Prior Conjugate Vaccine Exposure on LAV Efficacy

**Figure 3:**
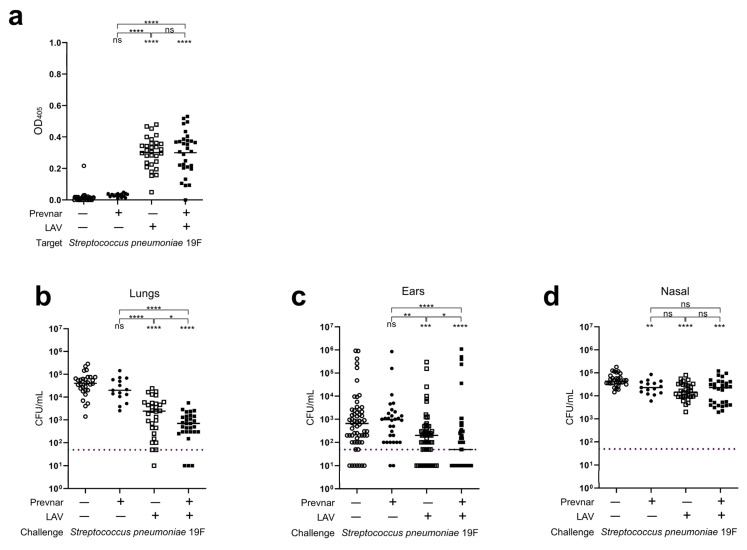
The protective efficacy of the LAV strain is not diminished with Prevnar-13 vaccination but rather demonstrates a significantly enhanced protective benefit. The mice were vaccinated with either a PBS vehicle control (N = 30; open circles), Prevnar-13 (N = 15; closed circles), LAV strain (N = 30; open squares), or Prevnar-13 followed by vaccination with the LAV strain (N = 30, closed squares). Following the final vaccination, the mice were challenged with a homologous serotype (19F). (**a**) Sera were collected prior to challenge, and IgG seroconversion was determined by ELISA against 19F. Each data point represents an individual mouse, and the bars represent the mean. The immunoreactivity of sera of the vaccinated mice were compared to that of the mice that received the PBS vehicle control or other vaccines via an unpaired *t*-test. (**b**–**d**) Twenty-four hours post-challenge with 19F, the bacterial burden in the lungs (**b**), ears (**c**), and nasal passage (**d**) of the mice was determined. Each data point represents an individual mouse (**b**,**d**) or each ear from individual mice (**c**), and the bars represent the median. The dashed line represents the limit of detection. The bacterial burden in each tissue of the vaccinated mice was compared to the burden in the tissues of the mice that received the PBS vehicle control or other vaccines via a non-parametric Mann–Whitney *t*-test. * *p* < 0.05, ** *p* < 0.01, *** *p* < 0.001, **** *p* < 0.0001, ns = non-significant.

Various formulations of pneumococcal conjugate vaccines have been approved and are recommended as part of the standard series of childhood vaccinations [85,86]. Widespread introduction of these vaccines resulted in dramatic alterations in the population structure of pneumococci and serotype replacement [87]. This strong selective pressure might be expected to diminish the protective efficacy of the LAV strain, as colonization duration and/or immune response might be dampened by pre-existing vaccine-based immunity. This was evaluated in a model whereby mice were vaccinated with a single vaccination of Prevnar-13 prior to vaccination with the LAV strain. Serum antibody titers demonstrated no significant differences in IgG levels against homologous (19F) (Figure 3a) and heterologous serotypes (7F) (Figure S3a) between the mice that received Prevnar-13 prior to the LAV strain and those that received the LAV strain alone. In the mice challenged with either 19F or 7F strains, the efficacy of protection of the LAV strain was not diminished in the mice that received Prevnar-13 but rather demonstrated enhanced protective benefit in the lungs and ears, with no difference in the nasal passage burden (Figure 3b–d; Figure S3b–d). These data indicate that the efficacy of the LAV strain is not reduced with prior vaccination and may synergize with Prevnar-13 to confer enhanced protection against mucosal infections such as AOM.

### 3.4. Efficacy of Polyvalent Vaccines to Protect Against AOM from Multiple Pathogens

#### 3.4.1. Engineering Polyvalent Vaccines

One challenge with utilizing vaccines to induce protection from AOM is the observation that multiple pathogens can cause AOM [88]. While *S. pneumoniae* is a leading cause of bacterial AOM, other bacterial pathogens, most notably *H. influenzae* and *M. catarrhalis*, are also highly prevalent causative agents of disease. This raises the distinct possibility that the deployment of a vaccine targeting a single pathogen may not result in an overall reduction in the clinical burden of AOM, despite promoting protection against one of the major pathogens. To address this pitfall, we sought to leverage the LAV strain as a platform to express non-native antigens on the cell surface to induce antibody responses and confer protection against multiple bacterial species.

**Figure 4:**
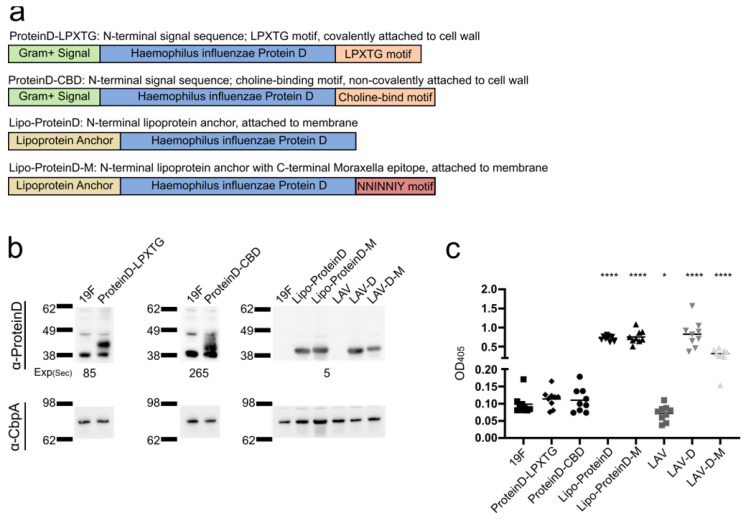
Novel platform for expressing non-native antigenic epitopes in *S. pneumoniae*. (**a**) Strategies for generating *S. pneumoniae* strains expressing antigenic epitopes of *H. influenzae* and *M. catarrhalis*. Three strategies were employed to anchor ProteinD to the cell surface, i.e., with C-terminal LPXTG motif (ProteinD-LPXTG), with C-terminal choline-binding motif (ProteinD-CBD), and with N-terminal lipoprotein anchor domain (Lipo-ProteinD). To generate a vaccine against all three otopathogens, both *H. influenzae* and *M. catarrhalis* epitopes were incorporated by expressing Lipo-ProteinD with an additional 23-amino-acid UspA epitope (“NNINNIY”) on the C-terminus. (**b**) The production of ProteinD in the cell lysates of *S. pneumoniae* expressing each ProteinD variant was measured via Western blot analysis using a polyclonal antibody against ProteinD. The strains included wild-type 19F, strains expressing each ProteinD variant, and the *ftsY* mutation in 19F (LAV), strain expressing Lipo-ProteinD (LAV-D), and strain expressing Lipo-ProteinD-M (LAV-D-M). The expression of ProteinD-LPXTG (51 kDa) and Protein-CBD (48 kDa) was detected upon longer exposure time than Lipo-ProteinD (42 kDa) and Lipo-ProteinD-M (45 kDa), and the exposure time (s) is listed for comparison. As a loading control, the samples were concurrently run and probed with an antibody against CbpA. (**c**) The anchoring of ProteinD on the *S. pneumoniae* cell surface was measured via whole-cell bacterial ELISA using a polyclonal antibody against ProteinD. As a control for differential binding to the plate, ProteinD immunoreactivity was normalized to immunoreactivity against LytA, a cell surface protein. Each data point represents a biological replicate, and the bars represent mean. The immunoreactivity of ProteinD-expressing strains and LAV strains was compared to that of 19F via an unpaired *t*-test. * *p* < 0.05, **** *p* < 0.0001.

*Streptococci* utilize several strategies to anchor proteins to the cell surface, including lipoprotein anchoring, LPXTG-mediated covalent attachment to the peptidoglycan, and non-covalent attachment via choline-binding domains which bind to the phosphocholine moieties that decorate the pneumococcal peptidoglycan [89,90]. We generated *S. pneumoniae* strains that expressed a highly conserved and immunogenic *H. influenzae* antigen (ProteinD) [72] with distinct anchoring strategies (Figure 4a). All three anchoring strategies were successfully generated with *S. pneumoniae* expressing the ProteinD variants as determined via Western blot analysis (Figure 4b) and ELISA (Figure 4c). The *S. pneumoniae* strain expressing lipoprotein-anchored ProteinD consistently demonstrated the greatest level of expression and, hence, was selected for advancement in in vivo efficacy studies.

We next sought to determine if the strain expressing Lipo-ProteinD could be modified further to generate a single vaccine strain that would confer protection against all three major otopathogens: *S. pneumoniae*, *H. influenzae*, and *M. catarrhalis*. In this strategy, we fused the 23 amino acid protective epitope shared by UspA1 and UspA2 from *M. catarrhalis* (“NNINNIY”) [73,91,92] to the C-terminus of the Lipo-ProteinD construct (Figure 4a). *S. pneumoniae* expressed and anchored the Lipo-ProteinD-M to the cell surface as determined via Western blot analysis (Figure 4b) and ELISA (Figure 4c). To attenuate *S. pneumoniae*, the ∆*ftsY* mutation was introduced into the strain expressing the *H. influenzae* Lipo-ProteinD and the strain expressing both *H. influenzae* and *M. catarrhalis* antigens, which are herein referred to as LAV-D and LAV-D-M, respectively. Of note, the level of ProteinD expressed was reduced in the LAV-D-M strain compared to the LAV-D strain. Attenuation of the vaccine strains was confirmed by determining in vitro growth, adhesion to epithelial cells, and bacterial burden in the lungs, ears, and nasal passage upon challenging the mice with the vaccine strains (Figure S2). All three LAV strains exhibited slower growth in vitro, with the LAV-D-M strain demonstrating the most pronounced delay (Figure 2a) and reduced autolysis. While the vaccine strains had similar levels of adhesion to respiratory epithelial cells compared to the wild-type 19F (Figure S2b) and retained robust colonization capacity (Figure S2c), all three LAV strains were attenuated with dramatically reduced burden in the lungs and ears compared to the wild-type (Figure S2c).

#### 3.4.2. Vaccination Efficacy

**Figure 5:**
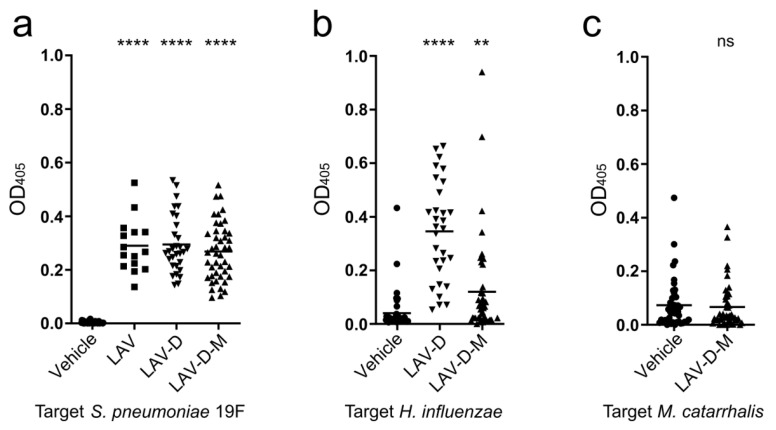
Vaccination with strains expressing non-native antigenic epitopes demonstrate seroconversion against multiple respiratory pathogens. (**a**–**c**) The mice were vaccinated with the LAV strain (squares), LAV-D strain (down triangles), LAV-D-M strain (up triangles), or PBS vehicle control (circles) and challenged with either *S. pneumoniae*, *H. influenzae*, or *M. catarrhalis*. Sera were collected prior to challenge, and IgG seroconversion in the vaccinated mice was determined by ELISA against 19F (**a**), *H. influenzae* (**b**), or *M. catarrhalis* (**c**) and included mice in all the challenge groups. Immunoreactivity against 19F was measured in the sera of the mice vaccinated with all the vaccine strains, against *H. influenzae* in the sera of the mice vaccinated with LAV-D or LAV-D-M, and against *M. catarrhalis* in the sera of the mice vaccinated with LAV-D-M; N = 15 for LAV, N = 30 for LAV-D, N = 45 for LAV-D-M and vehicle. Each data point represents an individual mouse, and the bars represent the mean. The immunoreactivity of the sera of the mice vaccinated with vaccine strains were compared to that of the mice that received the PBS vehicle control via an unpaired *t*-test. ** *p* < 0.01, **** *p* < 0.0001; ns = non-significant.

We next determined the efficacy of vaccination with the LAV-D and LAV-D-M strains to impart protection against the major otopathogens *S. pneumoniae*, non-typeable *H. influenzae*, and *M. catarrhalis*. Following intranasal vaccination, the levels of seroconversion was determined by ELISA (Figure 5). The mice vaccinated with LAV-D and LAV-D-M displayed significant IgG seroconversion against *S. pneumoniae* homologous 19F serotype and heterologous serotype 4 compared to the unvaccinated mice, at similar levels as the mice vaccinated with the LAV strain (Figure 5a, Supplemental Figure S4). Moreover, serum antibody titers against non-typeable *H. influenzae* were significantly increased compared to serum from the unvaccinated mice, with the level of protection in the mice vaccinated with LAV-D superseding that of LAV-D-M (Figure 5b). No notable differences were observed in serum titers against *M. catarrhalis* between the mice vaccinated with LAV-D-M and the unvaccinated mice (Figure 5c). However, the unvaccinated mice demonstrated some seroconversion to *M. catarrhalis* over the course of the vaccination regimen (baseline serum titers were ~0.028), suggesting the mice could have generated an antibody response to native flora similar to *M. catarrhalis*. These data indicate that the LAV-D and LAV-D-M strains, which add *H. influenzae* and *M. catarrhalis* antigens to the LAV strain, remained immunogenic against pneumococcus while conferring additional serum response against *H. influenzae*.

**Figure 6:**
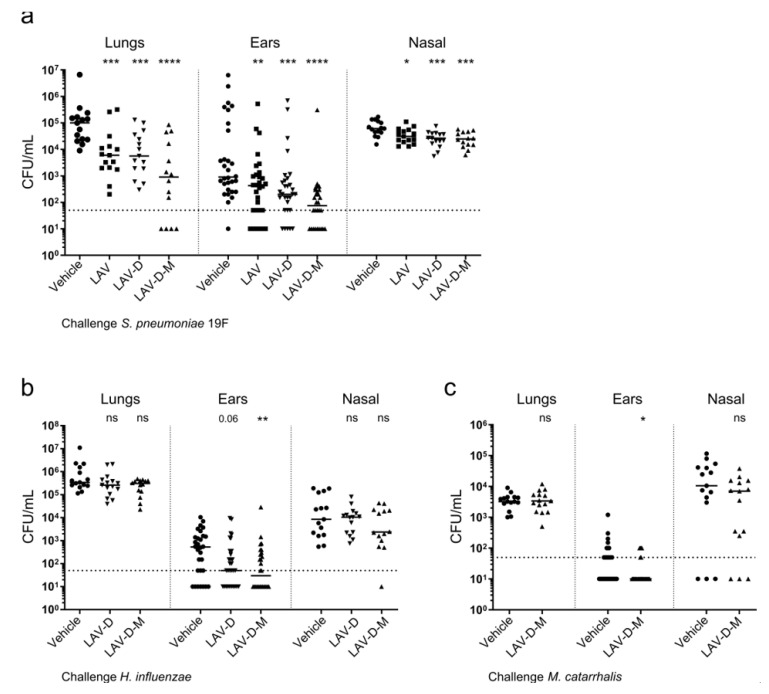
Vaccination with strains expressing non-native antigenic epitopes demonstrate protection against multiple respiratory pathogens. (**a**–**c**) The mice were vaccinated with the LAV strain (squares), LAV-D strain (down triangles), LAV-D-M strain (up triangles), or PBS vehicle control (circles) and challenged with either *S. pneumoniae*, *H. influenzae*, or *M. catarrhalis*; N = 15 for each challenge. The bacterial burden in the lungs, ears, and nasal passage of the vaccinated mice was determined for challenge with 19F (**a**), *H. influenzae* (**b**), or *M. catarrhalis* (**c**). All the mice challenged with *H. influenzae* or *M. catarrhalis* were pre-sensitized with poly (I:C) to enhance translocation to the ear. Each data point represents an individual mouse (lungs and nasal passage) or each ear from individual mice (ear), and the bars represent the median. The dashed line represents the limit of detection. For each challenge, the bacterial burden in each tissue of the vaccinated mice was compared to the burden in the tissues of the mice that received the PBS vehicle control via a non-parametric Mann–Whitney *t*-test. * *p* < 0.05, ** *p* < 0.01, *** *p* < 0.001, **** *p* < 0.0001. ns = non-significant.

The vaccinated mice were subsequently challenged with *S. pneumoniae*, non-typeable *H. influenzae*, or *M. catarrhalis*, and protection against bacterial burden in the lungs, ears, and nasal passage was determined (Figure 6). All the mice challenged with *H. influenzae* or *M. catarrhalis* were administered inflammatory stimulant poly (I:C) [77] prior to challenge to facilitate translocation into the middle ear (Figure S5). Vaccine strains incorporating the *H. influenzae* and *M. catarrhalis* epitopes (LAV-D and LAV-D-M) demonstrated protection against bacterial burden in the lungs and ears when challenged with *S. pneumoniae* compared to the unvaccinated vehicle control (Figure 6a), similar to the original LAV strain and in line with the observed seroconversion against *S. pneumoniae* in the mice vaccinated with all three LAV strains (Figure 5a). Notably, vaccination with the LAV-D-M strain demonstrated significantly enhanced protection against pneumococcal burden in the ears compared with vaccination with the LAV strain (*p* < 0.05). In addition to protection against *S. pneumoniae*, the mice vaccinated with LAV-D and LAV-D-M gained AOM protection against *H. influenzae* challenge, exhibiting reduced bacterial burden in the ears (Figure 6b). Of note, the burden in the ears of the mice vaccinated with LAV-D-M was reduced similarly to those vaccinated with LAV-D despite a lower seroconversion against *H. influenzae* in LAV-D-M compared to LAV-D (Figure 5b). The mice vaccinated with LAV-D-M also gained protection against AOM caused by *M. catarrhalis*, demonstrating reduced bacterial burden in the ears compared to the unvaccinated mice (Figure 6c), despite no observable difference in IgG seroconversion (Figure 5c). These data present a potentially viable approach for targeting all three major bacterial pathogens responsible for pediatric AOM through engineering the LAV strain to express non-native bacterial epitopes on the cell surface.

## 4. Discussion

*Streptococcus pneumoniae* is a human pathogen and a major cause of morbidity and mortality particularly in young children and elderly populations [93]. The development of a pneumococcus vaccine began in the early 1910s, with the first license obtained for the pneumococcal polysaccharide vaccine (PPV) in 1977 followed by the pneumococcal conjugated vaccine (PCV) in 2000 [94]. The PPV was updated from the original 14 serotypes in 1983 to cover 23 serotypes, while the PCV was updated from 7 serotypes to 13 in 2010 [95] and then to 20 serotypes in 2021 [96]. The PPV is modestly effective at preventing invasive disease in adults, although most studies were conducted in populations with high carriage rates [97]. The PCV is currently recommended as part of the standard series of childhood vaccinations and provides effective protection against invasive disease, including bacteremia and meningitis [98], with an unexpected secondary protective effect on pneumonia and otitis media through herd immunity [99,100]. However, this protection is serotype-specific, does not include acute bacterial sinusitis, and is modest against AOM and pneumonia [101,102]. Thus, a multitude of vaccine strategies have been employed to develop serotype-independent pneumococcal vaccines that can prevent the entire spectrum of pneumococcal associated disease, including sinusitis, otitis media, pneumonia, bacteremia, and meningitis.

We and others have proposed that live-attenuated pneumococcal vaccines could represent a viable alternative to polysaccharide-based vaccines, since desirable, heterosubtypic responses to pneumococcal proteins are generated in the context of a natural, mucosal colonization [38,39,40]. In this study, we further investigated a pneumococcal live vaccine platform based upon the deletion of *ftsY*, which confers protective immunity against both invasive disease and AOM in a serotype-independent manner. Our data suggest that there would likely be minimal risk of genetic reversion of the LAV strain, as the deletion of *ftsY* conferred a significant impairment to uptake and recombine genomic DNA. The underlying mechanism for the reduced competence of the *ftsY* deletion is unclear but could be due to impaired assembly of the competence machinery into the pneumococcal cell membrane given the role of the SRP pathway for the appropriate targeting of integral membrane proteins [103]. The *ftsY* mutant also demonstrated reduced growth kinetics, suggesting that the deleterious fitness cost of obtaining such a mutation may also decrease its propensity for horizontal transfer. In many strain backgrounds, no recoverable *ftsY* mutants were recovered despite the transformed strain being competent. We postulate that this is due to strain-specific gene essentiality of the SRP pathway, restricting the transfer of this mutation to strain backgrounds permissive for deletion. This agrees with previous studies investigating gene essentiality across multiple strains of pneumococcus, whereby it was also observed that *ftsY* gene deletion was only tolerated in a subset of strains tested [104]. Further underscoring the genetic stability of the LAV strain, no reversion to the wild-type genotype was observed despite in vivo passaging.

Unlike traditional protein-based or polysaccharide vaccines, a potential limitation of LAVs is the interference by pre-existing immunity which could enhance clearance of the LAV and thus may adversely impact vaccine efficacy. For the pneumococcus, this could result from prior colonization by pneumococcal strains or immunity conferred by previous PCV vaccination. Prior colonization with strains harboring either a homologous or heterologous genotype/serotype did not significantly impact the protection imparted by vaccination with the LAV strain. Of note, it is unclear if the LAV strain colonized the nasal passage of the mice that were previously colonized as well as those that were non-colonized. It is unlikely that prior colonization would reduce LAV colonization to such an extent as to prevent an immune response against the vaccine strain. Previous studies have indicated that prior colonization can contribute to early clearance of secondary colonizing strains [105]. However, the secondary colonizing strain can initially colonize the nasal passage and persist up to 14 days [106,107,108]. The LAV strain itself is cleared after seven days [39], suggesting that the LAV strain would clear in the same time period regardless of prior colonization. Moreover, colonizing strains can induce antibody production in the murine model [107,109], and this may explain the enhanced seroconversion observed in the vaccinated mice in the context of prior colonization compared to a non-colonized control (Figure 2a). These data agree with prior observations that repeated exposure events confer heightened immune protection against pneumococcus [105,110]. As observed with prior colonization, the mice prevaccinated with Prevnar-13 demonstrated a similar or synergistic protective effect on the development of AOM. These data indicate that pre-existing immunity via either colonization or vaccination does not inhibit the protective capacity of the LAV strain and may enhance the protection conferred by the vaccine. 

A main limitation of vaccines targeting any one etiological agent of AOM is the observation that multiple bacterial pathogens are responsible for the disease, making it unclear if a vaccine targeting any one organism would be successful in reducing overall clinical disease burden. To address this potential limitation, we engineered an LAV strain that expressed *H. influenzae* ProteinD via multiple anchoring mechanisms, indicating that novel epitopes could be introduced and expressed in the pneumococcus backbone. While all anchoring strategies were successful, the incorporation of a lipoprotein anchor, in a similar manner to how ProteinD is anchored in *H. influenzae* [111], yielded the highest level of protein expression and anchoring to the pneumococcal cell surface. This suggests that the exploration and validation of multiple routes of anchoring may be required to determine the optimal strategy for the surface expression of novel proteins. 

We utilized the lipoprotein anchoring strategy to explore broadening the protective capacity of the LAV strain against multiple bacterial species. To further broaden pathogen coverage to include all three major AOM pathogens, we generated an LAV strain expressing a highly conserved epitope from *M. catarrhalis* UspA1/2 fused to Lipo-ProteinD. This epitope was chosen due to previous studies demonstrating the protective efficacy of monoclonal antibodies targeting this conserved antigen [91,92]. We were able to successfully integrate and express the Lipo-ProteinD and *Moraxella* epitope in the LAV strain background. These data indicate that a single LAV strain can be engineered to serve as an antigen presentation system for diverse bacterial antigens. All three vaccine strains conferred significant protection against pneumococcal pathogenesis, including pneumonia and AOM. Interestingly, vaccination with the LAV-D-M strain demonstrated significantly enhanced protection against pneumococcal burden in the ears compared to vaccination with the LAV strain. The mechanism behind this phenomenon is unclear and likely multifactorial. *H. influenzae* ProteinD, encoded by *glpQ*, is a glycerophosphodiester phosphodiesterase that catabolizes glycerophosphorylcholine (GCP) to free choline [112,113]. *S. pneumoniae* has one or two *glpQ* orthologs depending on the serotype, with the *glpQ2* ortholog resembling that of *H. influenzae*. The deletion of *glpQ2* in *S. pneumoniae* results in reduced choline on the cell surface, a lack of autolysis, reduced adhesion and cytotoxicity in cell lines, and reduced bacterial load in murine nasal passages and lungs [114]. While *glpQ2* is present in *S. pneumoniae* 19A and Taiwan-19F strains, it is absent in the BHN97 19F strain used in this study. The expression of *H. influenzae* ProteinD in the BHN97 background may provide a means for BHN97 to obtain free choline from GCP. This, in turn, may increase phosphorylcholine in the cell wall and enhance binding to epithelial cells and colonization [115,116,117,118]. In addition to conferring protection against pneumococcal pathogenesis, the LAV strains expressing ProteinD conferred protection against *H. influenzae* challenge. Of note, the trivalent LAV-D-M strain was less effective than the LAV-D strain in seroconversion against *H. influenzae*. This was likely due to the reduced level of ProteinD expressed in the LAV-D-M strain compared to the LAV-D strain or perhaps the additional Moraxella epitope on the C-terminus end of ProteinD reduced its immunogenicity. Both LAV-D and LAV-D-M strains were protective against AOM caused by *H. influenzae*, while demonstrating no significant protective capacity in terms of reduction in bacterial load in the lungs, in line with previous ProteinD vaccine studies [119]. Similarly, vaccine strains expressing both *H. influenzae* and *M. catarrhalis* antigens protected against AOM caused by both otopathogens but had no significant impact on bacterial burden in the lungs.

## 5. Conclusions

Taken together, these data demonstrate a novel platform for engineering attenuated vaccine strains to express novel surface epitopes that are capable of inducing protection against multiple pathogens. 

## Data Availability

The raw data values underlying the graphed data and the reported means presented in both the main text and Supplementary Materials as well as any additional relevant information about the data are available upon request directly from the corresponding author.

## Supplementary Materials

The following supporting information can be downloaded at https://www.mdpi.com/article/10.3390/vaccines12121432/s1, File S1: The original Western blot figures; Table S1: Strains used in this study; Table S2: Primers used to generate LAV, LAV-D, and LAV-D-M; Table S3: ProteinD Protein Sequences; Figure S1: LAV strain confers protection against multiple pneumococcal serotypes; Figure S2: LAV strains are characterized by a slower growth rate and reduced virulence; Figure S3: The protective efficacy of the LAV strain is not diminished with Prevnar-13 vaccination; Figure S4: Vaccination with strains expressing non-native antigenic epitopes demonstrate seroconversion against heterologous S. pneumoniae serotype 4; Figure S5: Poly (I:C) treatment prior to challenge enhances localization to respiratory tissues. References [65,120,121,122,123] are cited in the Supplementary Materials.
