# Supplementary material for: A Trivalent Live Vaccine Elicits Cross-Species Protection Against Acute Otitis Media in a Murine Model
Source: Vaccines (Basel). 2024 Dec 19;12(12):1432. doi: 10.3390/vaccines12121432 (PMC11728825; doi:10.3390/vaccines12121432)
Supplement: Supplementary file 1 [file vaccines-12-01432-s001.zip › File S1. The original Western blot figures.pdf]

Blot 1

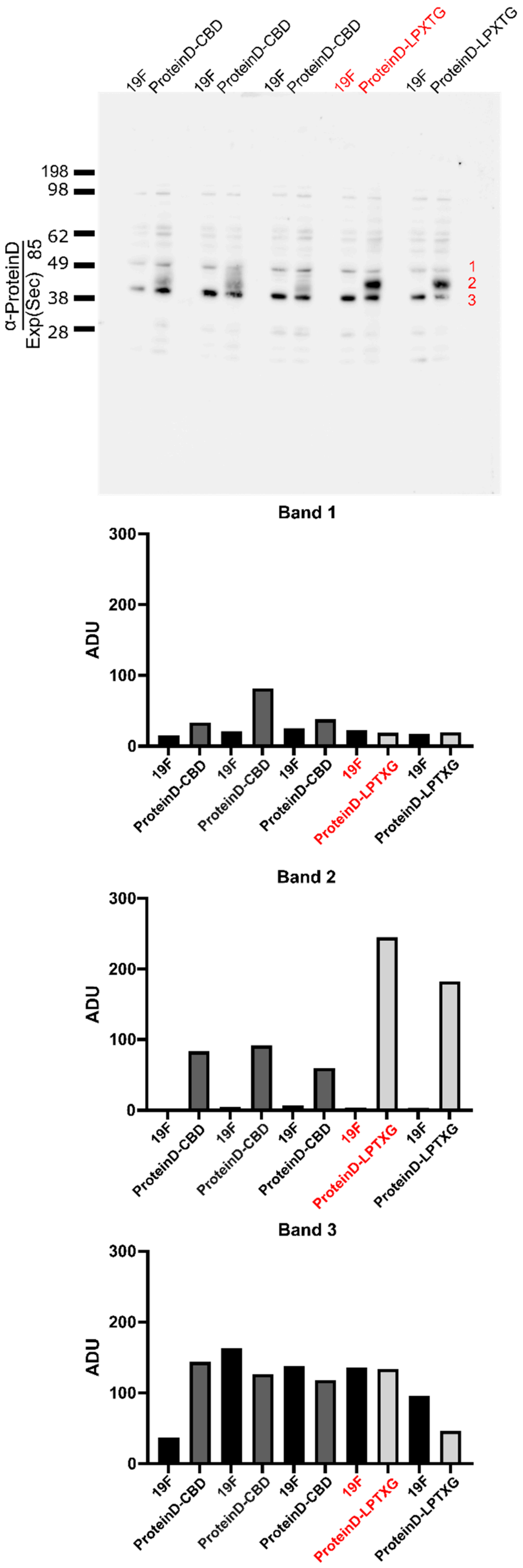

19F
ProteinD-CBD
19F
ProteinD-CBD
19F
ProteinD-CBD
19F
ProteinD-LPXTG
19F
ProteinD-LPXTG
198
98
62
49
38
28
α-ProteinD
Exp(Sec) 85
1
2
3
Band 1
Band 2
Band 3
ADU
300
200
100
0

Blot 1

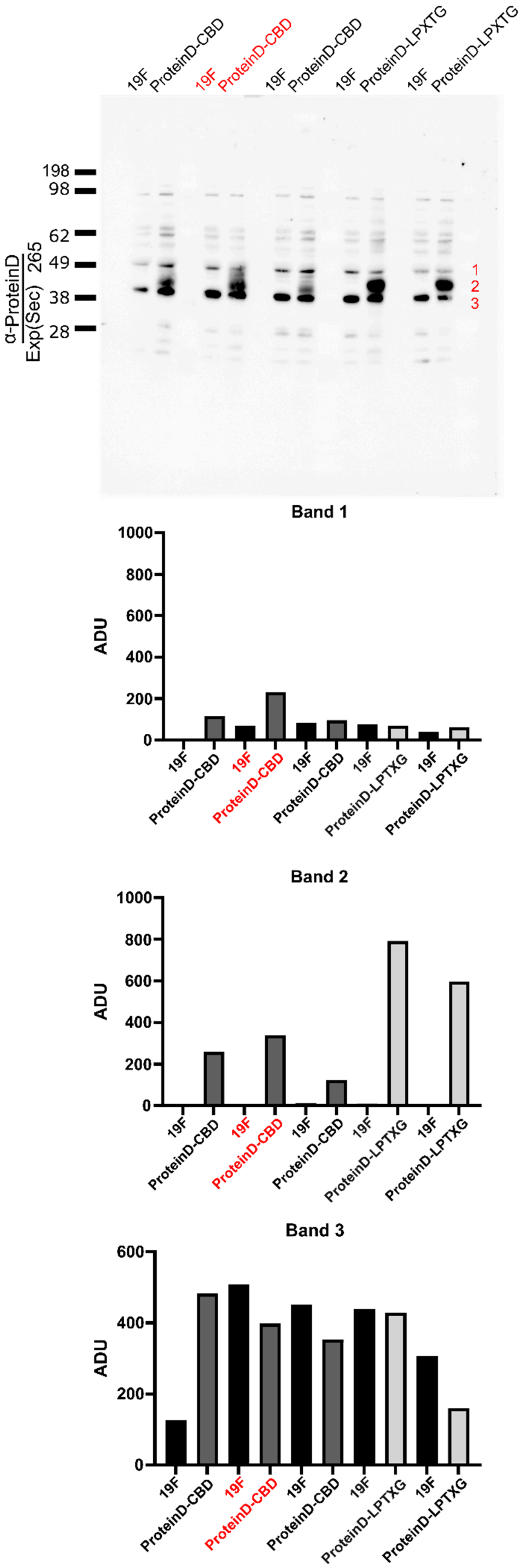

19F
ProteinD-CBD
19F
ProteinD-CBD
19F
ProteinD-CBD
19F
ProteinD-LPXTG
19F
ProteinD-LPXTG
198
98
62
49
38
28
α-ProteinD
Exp(Sec) 265
1
2
3
Band 1
ADU
Band 2
Band 3

Blot 2

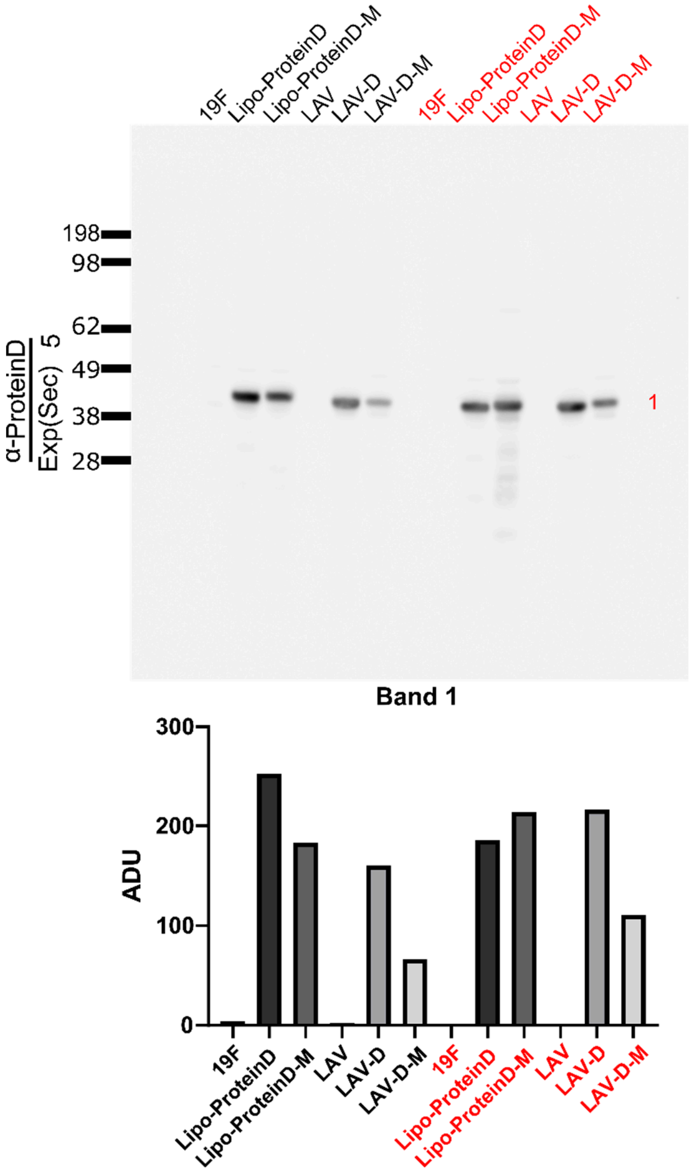
19F
Lipo-ProteinD
Lipo-ProteinD-M
LAV
LAV-D
LAV-D-M
19F
Lipo-ProteinD
Lipo-ProteinD-M
LAV
LAV-D
LAV-D-M
198
98
62
49
38
28
α-ProteinD
Exp(Sec) 5
1
Band 1
300
200
100
0
ADU

Blot 3

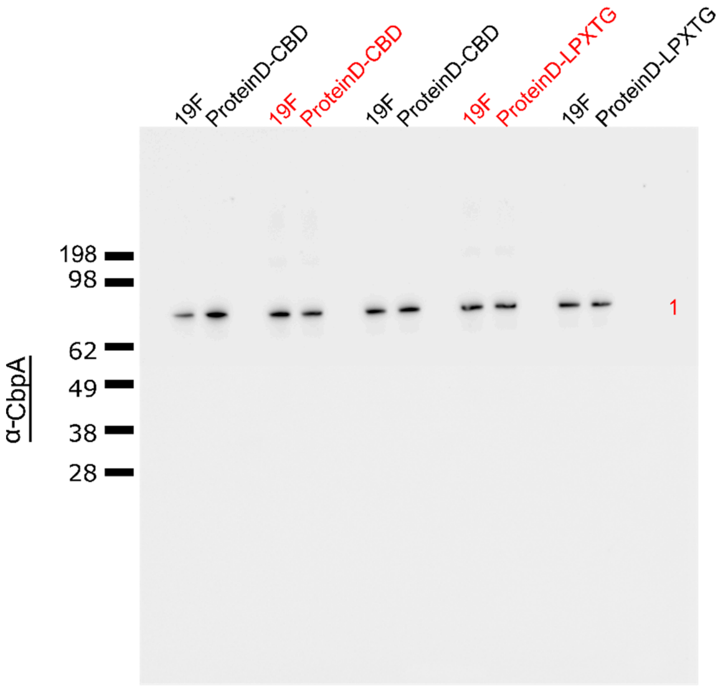
19F
ProteinD-CBD
19F
ProteinD-CBD
19F
ProteinD-CBD
19F
ProteinD-LPXTG
19F
ProteinD-LPXTG
198
98
62
49
38
28
α-CbpA
1

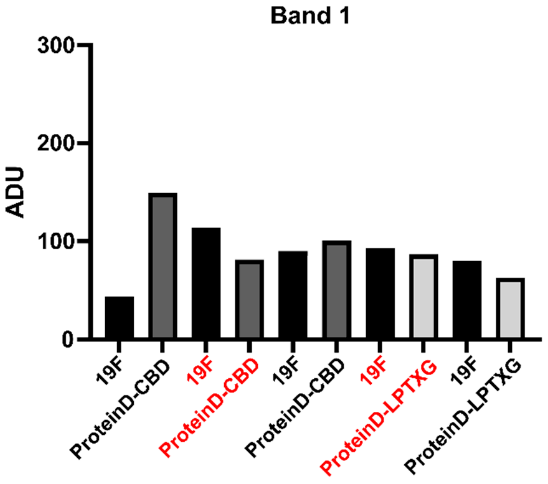
Band 1
ADU
300
200
100
0
19F
ProteinD-CBD
19F
ProteinD-CBD
19F
ProteinD-CBD
19F
ProteinD-LPTXG
19F
ProteinD-LPTXG

Blot 4

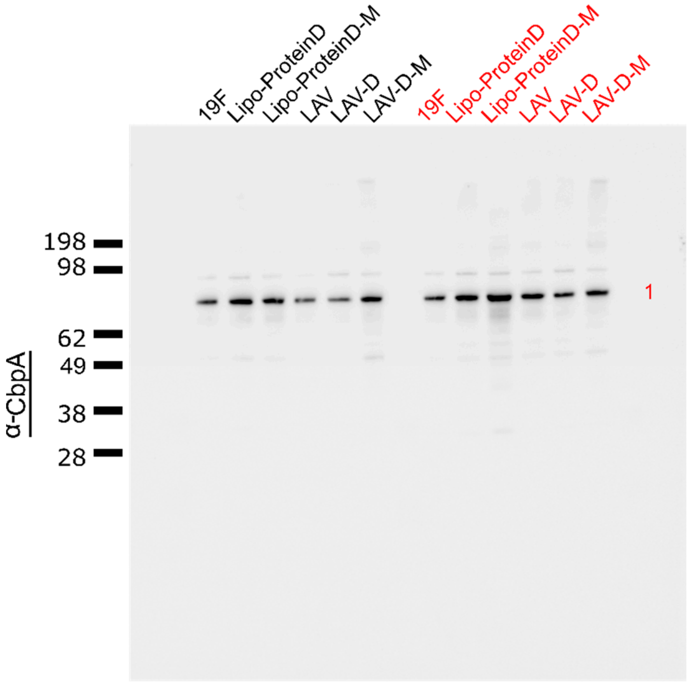

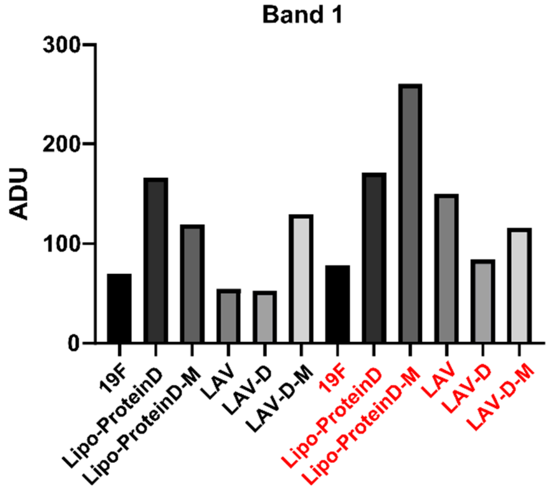

Unedited Blots used in Figure 4. Production of ProteinD in cell lysates of *S. pneumoniae* was measured via western blot analysis using a polyclonal antibody against ProteinD (Blots 1 and 2). As a loading control, samples were concurrently run and probed with an antibody against CbpA (Blots 3 and 4). Blot 1 and 3 included lysates of wild-type 19F and strains expressing ProteinD-CBD and ProteinD-LPXTG. Blot 2 and 4 included lysates of 19F, Lipo-ProteinD, Lipo-ProteinD-M, and the *ftsY* mutation in 19F (LAV), strain expressing Lipo-ProteinD (LAV-D), and strain expressing Lipo-ProteinD-M (LAV-D-M). Multiple biological replicates were run on each blot and the replicate set with the most similar CbpA levels in all samples was selected for publication (red text). Expression of ProteinD-LPXTG (51 kDa) and Protein-CBD (48 kDa) was detected upon longer exposure time than Lipo-ProteinD (42 kDa) and Lipo-ProteinD-M (45 kDa) and exposure time (sec) is listed. Below each blot is the densitometry analysis (reported as arbitrary densitometry units) for the bands listed in red numbers on the corresponding blot.
