# Supplementary material for: A Trivalent Live Vaccine Elicits Cross-Species Protection Against Acute Otitis Media in a Murine Model
Source: Vaccines (Basel). 2024 Dec 19;12(12):1432. doi: 10.3390/vaccines12121432 (PMC11728825; doi:10.3390/vaccines12121432)
Supplement: Supplementary file 1 [file vaccines-12-01432-s001.zip › vaccines-3325062-supplementary.pdf]

Supplemental Table S1. Strains used in this study.

Strain	Strain Description	Source
19F (BHN97)	BHN97; *Streptococcus pneumoniae* Serotype 19F	[1]
19Fx (BHN97x)	Bioluminescent BHN97 with Tn4001 *luxABCDE*; Kan^R; *S. pneumoniae* serotype 19F	[2]
7Fx (BHN54x)	Bioluminescent BHN54 with Tn4001 *luxABCDE*; Kan^R; *S. pneumoniae* serotype 7F	[1]
Haemophilus influenzae	Non-typeable 86-028NP	[3]
Moraxella catarrhalis	O35E	[3]
LAV	Internal deletion of *ftsY* (Sp1244) *ftsy*::PhunSweetErm in BHN97; Erm^R	This study
BHN97 CEPΩPhunSweetErm	Insertion of PhunSweetErm into CEP locus in BHN97; Erm^R	This study
BHN97 CEPΩ*proteinD-LPXTG*	Insertion of *proteinD* with C-terminal LPXTG motif into CEP locus under P3 promoter in BHN97	This study
BHN97 CEPΩ*proteinD-CBD*	Insertion of *proteinD* with C-terminal CBD motif into CEP locus under P3 promoter in BHN97	This study
BHN97 CEPΩ*lipo-proteinD*	Insertion of *proteinD* with N-terminal lipoanchor motif into CEP locus under P3 promoter in BHN97	This study
BHN97 CEPΩ*lipo-proteinD-M*	Insertion of *proteinD* with N-terminal lipoanchor motif and C-terminal NNINNIY epitope tag into CEP locus under P3 promoter in BHN97	This study
LAV-D	Insertion of *proteinD* with N-terminal lipoanchor motif into CEP locus under P3 promoter in BHN97; Internal deletion of *ftsY* (Sp1244) *ftsy*::PhunSweetErm; Erm^R	This study
LAV-D-M	Insertion of *proteinD* with N-terminal lipoanchor motif and C-terminal NNINNIY epitope tag into CEP locus under P3 promoter in BHN97; Internal deletion of *ftsY* (Sp1244) *ftsy*::PhunSweetErm; Erm^R	This study
4::4	*S. pneumoniae* TIGR4 strain with capsule locus replaced with type 4 capsule locus; K56T *rpsL* mutation to confer $Strep^R$	[4]
4::19F	*S. pneumoniae* TIGR4 strain with capsule locus replaced with type 19F capsule locus; K56T *rpsL* mutation to confer $Strep^R$	This study
19F::19F	*S. pneumoniae* BHN97 strain with capsule locus replaced with type 19F capsule locus; K56T *rpsL* mutation to confer $Strep^R$	This study
19F::4	*S. pneumoniae* BHN97 strain with capsule locus replaced with type 4 capsule locus; K56T *rpsL* mutation to confer $Strep^R$	This study
BHN97 Tnseq	Tn-Seq library generated in BHN97; $Spec^R$	[5]
Serotype 4	*S. pneumoniae* TIGR4	
Serotype 2	*S. pneumoniae* D39	
Serotype 22F	*S. pneumoniae* CDC isolate ABC A1	CDC
Serotype 33F	*S. pneumoniae* CDC isolate ABC A2	CDC
Serotype 15A	*S. pneumoniae* CDC isolate ABC A3	CDC
Serotype 6C	*S. pneumoniae* CDC isolate ABC A4	CDC
Serotype 10A	*S. pneumoniae* CDC isolate ABC A17	CDC
Serotype 35B	*S. pneumoniae* CDC isolate ABC A31	CDC
Serotype 18C	*S. pneumoniae* CDC isolate ABC A33	CDC
Serotype 6B	*S. pneumoniae* CDC isolate ABC A35	CDC
Serotype 9N	*S. pneumoniae* CDC isolate ABC A37	CDC
Serotype 6A	*S. pneumoniae* CDC isolate ABC A43	CDC

Supplemental Table S2. Primers used to generate LAV, LAV-D, and LAV-D-M. For primers Ftsy_Up_R and CEP_Up_PS_R, nucleotides in bold are overlapping sequence of 5' of PhunSweet. For primers Ftsy_Down_F and CEP_Down_PS_F, nucleotides in bold are overlapping sequence of 3' of *erm*. For primers CEP_Up_D_R and CEP_Down_D_F, nucleotides in bold are overlapping sequence of 5' of P3 and 3' of terminator from Genscript synthesized constructs. For primer Lipo-ProteinD_2_F, nucleotides in bold are overlapping sequence of 3' of the first fragment of *lipo-proteinD*. For primers Lipo-ProteinD_1_F and Lipo-ProteinD_2_R, nucleotides in bold are overlapping sequence of 3' of upstream flank of CEP site and 5' of downstream flank of CEP site, respectively, and were included to enhance overlap during SOE PCR. For primers Lipo-ProteinD_N_F and CEP_Down_N_F, nucleotides in bold are overlapping sequence of 5' and 3' ends of NNINNIY ultramer fragment.

Name	Sequence
Phunsweet_F	CAATTAACTTTACAAATTCCCACTATTAAGG
Erm_R	CCAAATTTACAAAAGCGACTC
FtsY_Up_F	CTTGGTATCACAATGGATCAGGTCATG
FtsY_Up_R	**CTTAATAGTGGGAATTTGTAAAGTTAATTG**CTGACTTTCTTCAACTGTGTCTTGGATTTC
FtsY_Down_F	**GAGTCGCTTTTGTAAATTTGG**GTACAGGCCAAAGAATTTTCGAAAATCACAC
FtsY_Down_R	CTTGGTAAGGAAATGATCCAAAGCATC
CEP_Up_F	GCAAATCTTTGGCTTCTTGTTCAAATTTTC
CEP_Up_PS_R	**ATAGTGGGAATTTGTAAAGTTAATTG**GATCTGGTGTCTCAGTCTTTTATTTCTTGCG
CEP_Down_PS_F	**GAGTCGCTTTTGTAAATTTGG**TAATTCCCATAAAAATTGACATGGAAATTATAAA
CEP_Down_R	ATGTCTGAAAAATTAGTAGAAATCAAAGATTTAGAAATTTC
CEP_Up_D_R	**CATAAGGTAAACTTTTGAGTGCTTT**GATCTGGTGTCTCAGTCTTTTATTTCTTGCG
CEP_Down_D_F	**CCCTGACAGGGCGCGGTTTTTTTTT**TAATTCCCATAAAAATTGACATGGAAATTATAAA
ProteinD_F	AAAGCACTCAAAAGTTTACCTTATGGGTGC
ProteinD_R	AAAAAAAAACCGCGCCCTGTCAGGG
Lipo-ProteinD_1_F	**GAAATAAAAGACTGAGACACCAGATC**AAAGCACTCAAAAGTTTACCTTATGGGTGC
Lipo-ProteinD_1_R	CAAAGATTGAATTTCCTTCAAAGTAAAATC
Lipo-ProteinD_2_F	**TTTTACTTTGAAGGAAATTCAATCTTTG**GAAATGACAGAAAATTTTGAAACTAAAGATGG
Lipo-ProteinD_2_R	**TAATTTCCATGTCAATTTTTATGGGAATTA**AAAAAAAAACCGCGCCCTGTCAGGG
NNINNIY_Ultra_F	AACAATATAAACAACATTTATGAATTGGCGCAACAGCAAGATCAGCACTCTTCAGACATAAAAACGCTA
NNINNIY_Ultra_R	TAGCGTTTTTATGTCTGAAGAGTGCTGATCTTGCTGTTGCGCCAATTCATAAATGTTGTT TATATTGTT
NNINNIY_F	AACAATATAAACAACATTTATGAATTGGCG
NNINNIY_R	TAGCGTTTTTATGTCTGAAGAGTGCTG
Lipo-ProteinD_N_R	**CGCCAATTCATAAATGTTGTTTATATTGTT**-TTTAATACCTTTCAAAAATTCAACTCCAGT
CEP_Down_N_F	**ATCAGCACTCTTCAGACATAAAAACGCTA**GGATCCCTCCAGTAACTCGAGAAAAAAAAAC
FtsY_Outer_F	GTAGCGAGTTTGGAAGATATCACAG
FtsY_Outer_R	CACAATAAGCTCTTCTTCTTGTCTATGG
FtsY_Inner_F	GAAACTGTTCAGGAAAAATATGACCGCAGTCTTAAG
FtsY_Inner_R	CAACTCAGCCATAAGGTTATCCTTATTTTGCAGAC

Supplemental Table S3. ProteinD Protein Sequences.

ProteinD-CBD	MKHEKQQRFSIRKYAVGAASVLIGFAFQAQTVAADGVTSSHSSNMANTQMKSDKIIIA<br>HRGASGYLPEHTLESKALAFAQQADYLEQDLAMTKDGRLVVIHDHFLDGLTDVAKKF<br>PHRHRKDGRYYVIDFTLKEIQSLEMTENFETKDGKQAQVYPNRFPLWKSHFRIHTFED<br>EIEFIQGLEKSTGKKVGIYPEIKAPWFHHQNGKDIAAETLKVLKKYGYDKKTDMVYLQT<br>FDFNELKRIKTELLPQMGMDLKLVQLIAYTDWKETQEKDPKGYWVNYNYDWMFKPG<br>AMAEVVKYADGVGPGWYMLVNKEESKPDNIVYTPLVKELAQYNVEVHPYTVRKDALP<br>EFFTDVNQMYDALLNKSGATGVFTDFPDTGVEFLKGIKLWYYLNESGSMATGWVKDK<br>GLWYYLNESGSMATGWVKDKGLWYYLNESGSMATGWVKDKGGSLQ-
ProteinD-LPXTG	MKHEKQQRFSIRKYAVGAASVLIGFAFQAQTVAADGVTSSHSSNMANTQMKSDKIIIA<br>HRGASGYLPEHTLESKALAFAQQADYLEQDLAMTKDGRLVVIHDHFLDGLTDVAKKF<br>PHRHRKDGRYYVIDFTLKEIQSLEMTENFETKDGKQAQVYPNRFPLWKSHFRIHTFED<br>EIEFIQGLEKSTGKKVGIYPEIKAPWFHHQNGKDIAAETLKVLKKYGYDKKTDMVYLQT<br>FDFNELKRIKTELLPQMGMDLKLVQLIAYTDWKETQEKDPKGYWVNYNYDWMFKPG<br>AMAEVVKYADGVGPGWYMLVNKEESKPDNIVYTPLVKELAQYNVEVHPYTVRKDALP<br>EFFTDVNQMYDALLNKSGATGVFTDFPDTGVEFLKGIKLPETGTHDSAELVVAGLMST<br>LAAYGLTKRKEDGSLQ-
Lipo-ProteinD	MKLKTLALSLLAAGVLAGCSSHSSNMANTQMKSDKIIIAHRGASGYLPEHTLESKALAF<br>AQQADYLEQDLAMTKDGRLVVIHDHFLDGLTDVAKKFPHRHRKDGRYYVIDFTLKEIQ<br>SLEMTENFETKDGKQAQVYPNRFPLWKSHFRIHTFEDEIEFIQGLEKSTGKKVGIYPEI<br>KAPWFHHQNGKDIAAETLKVLKKYGYDKKTDMVYLQTFDFNELKRIKTELLPQMGMD<br>LKLVQLIAYTDWKETQEKDPKGYWVNYNYDWMFKPGAMAEVVKYADGVGPGWYML<br>VNKEESKPDNIVYTPLVKELAQYNVEVHPYTVRKDALPEFFTDVNQMYDALLNKSGAT<br>GVFTDFPDTGVEFLKGIKGSLQ-
Lipo-ProteinD-M	MKLKTLALSLLAAGVLAGCSSHSSNMANTQMKSDKIIIAHRGASGYLPEHTLESKALAF<br>AQQADYLEQDLAMTKDGRLVVIHDHFLDGLTDVAKKFPHRHRKDGRYYVIDFTLKEIQ<br>SLEMTENFETKDGKQAQVYPNRFPLWKSHFRIHTFEDEIEFIQGLEKSTGKKVGIYPEI<br>KAPWFHHQNGKDIAAETLKVLKKYGYDKKTDMVYLQTFDFNELKRIKTELLPQMGMD<br>LKLVQLIAYTDWKETQEKDPKGYWVNYNYDWMFKPGAMAEVVKYADGVGPGWYML<br>VNKEESKPDNIVYTPLVKELAQYNVEVHPYTVRKDALPEFFTDVNQMYDALLNKSGAT<br>GVFTDFPDTGVEFLKGIKNINNIYELAQQQDQHSSDIKTLGSLQ-
His-ProteinD	MGSSHHHHHHSSGLVPRGSHMSSHSSNMANTQMKSDKIIIAHRGASGYLPEHTLESK<br>ALAFAQQADYLEQDLAMTKDGRLVVIHDHFLDGLTDVAKKFPHRHRKDGRYYVIDFTL<br>KEIQSLEMTENFETKDGKQAQVYPNRFPLWKSHFRIHTFEDEIEFIQGLEKSTGKKVGI<br>YPEIKAPWFHHQNGKDIAAETLKVLKKYGYDKKTDMVYLQTFDFNELKRIKTELLPQM<br>GMDLKLVQLIAYTDWKETQEKDPKGYWVNYNYDWMFKPGAMAEVVKYADGVGPG<br>WYMLVNKEESKPDNIVYTPLVKELAQYNVEVHPYTVRKDALPEFFTDVNQMYDALLN<br>KSGATGVFTDFPDTGVEFLKGIK-

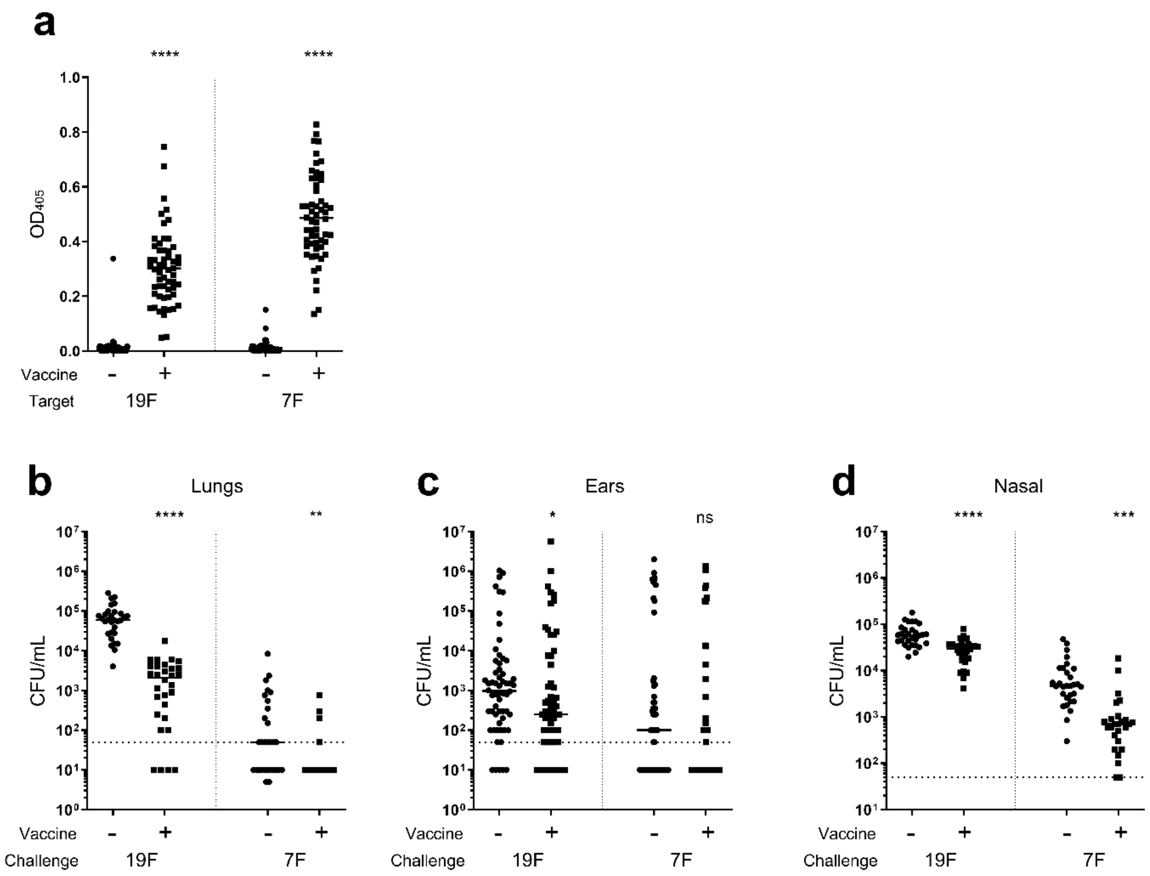

Supplemental Figure S1. LAV strain confers protection against multiple pneumococcal serotypes. (**a**–**d**) Mice were vaccinated with LAV strain (+) or PBS vehicle control (-) and challenged with either a homologous serotype (19F) or a heterologous serotype (7F); N=30. (**a**) Sera was collected prior to challenge and IgG seroconversion in mice was determined by ELISA against 19F or 7F and included mice in all challenge groups. Each data point represents an individual mouse and bars represent mean. Immunoreactivity of sera from mice vaccinated with the LAV strain was compared to that of mice that received the PBS vehicle control via unpaired t-test. (**b**–**d**) 24 hours post-challenge, bacterial burden in the lungs (**b**), ears (**c**), and nasal passage (**d**) of vaccinated mice was determined for each challenge. Each data point represents an individual mouse (**b,d**) or each ear from individual mice (**c**) and bars represent median. Dashed line represents limit of detection. For each challenge, bacterial burden in each tissue from mice vaccinated with LAV strain was compared to those receiving the PBS vehicle control via non-parametric Mann-Whitney t-test. *p<0.05, **p<0.01, ***p<0.001, ****p<0.0001.

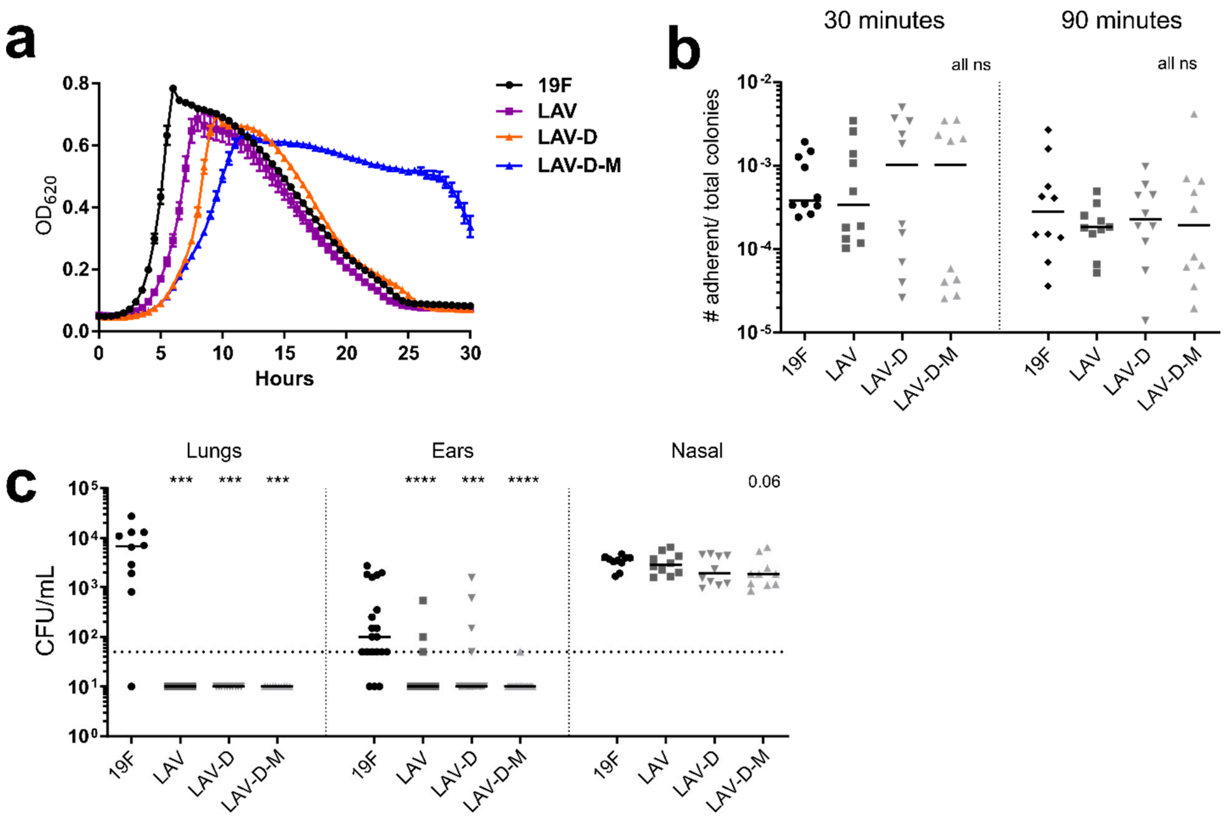

Supplemental Figure S2. LAV strains are characterized by slower growth rate and reduced virulence. (**a**) Vaccine strains were assayed for growth *in vitro* in semi-chemically defined media C+Y. Each strain was grown in liquid media and frozen stocks were made in triplicate. Each stock was diluted in a 96-well plate in triplicate and absorbance was monitored every 30 minutes for 30 hours using Biotek Cytation plate reader; this was repeated one time. Growth curves of the LAV strains were compared to that of wild-type 19F via two-way ANOVA, repeated measures; all had significance ****$p<0.0001$. (**b**) Adherence of the vaccine strains to epithelial cells was measured by adherence assays to A549 cells and the number of adherent colonies after 30- and 60-minutes incubation was determined. Adherence of LAV strains was compared to that of 19F via non-parametric Mann-Whitney t-test; no significant difference was observed. (**c**) The attenuation of the three LAV strains was confirmed by challenging mice with each of the LAV strains and wild-type 19F and determining bacterial burden in the lungs, ears, and nasal passages. Each data point represents an individual mouse (lungs, nasal passage) or each ear from individual mice (ear) and bars represent median. Dashed line represents limit of detection. Bacterial burden in mice challenged with the LAV strains was compared to that of mice challenged with 19F via non-parametric Mann-Whitney t-test. ***$p<0.001$, ****$p<0.0001$. There was no significant difference in bacterial burden in the lungs, ears, and nasal passage between the LAV-D or LAV-D-M strain when compared to the LAV via non-parametric Mann-Whitney t-test. Six of the mice challenged with 19F and LAV were used for *in vivo* passaging and the data shown here was also used in Figure 1 (Passage 1).

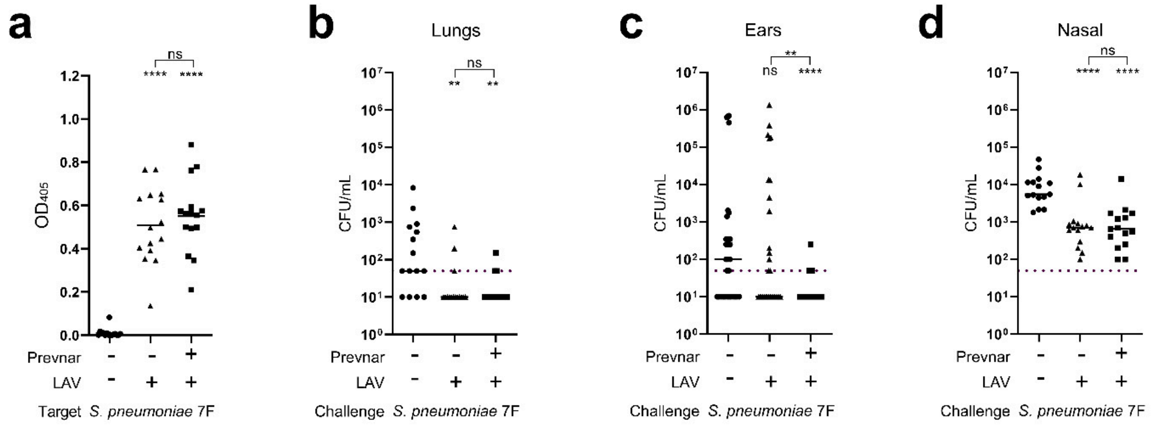

Supplemental Figure S3. The protective efficacy of the LAV strain is not diminished with Prevnar-13 vaccination. Mice were vaccinated with either PBS vehicle control, LAV strain, or Prevnar-13 followed by vaccination with the LAV strain; N=15. Following the final vaccination, mice were challenged with a heterologous serotype (7F). (**a**) Sera was collected prior to challenge and IgG seroconversion in vaccinated mice was determined by ELISA against 7F. Each data point represents an individual mouse and bars represent mean. Immunoreactivity of sera of mice vaccinated with vaccine strains were compared to that of mice that received the PBS vehicle control or other vaccines via unpaired t-test. (**b–d**) 24 hour post-challenge, bacterial burden in the lungs (**b**), ears (**c**), and nasal passage (**d**) of mice was determined. Each data point represents an individual mouse (**b,d**) or each ear from individual mice (**c**) and bars represent median. Dashed line represents limit of detection. Bacterial burden in each tissue of vaccinated mice was compared to the burden in tissues of mice receiving the PBS vehicle control or other vaccines via non-parametric Mann-Whitney t-test. **p<0.01, ****p<0.0001.

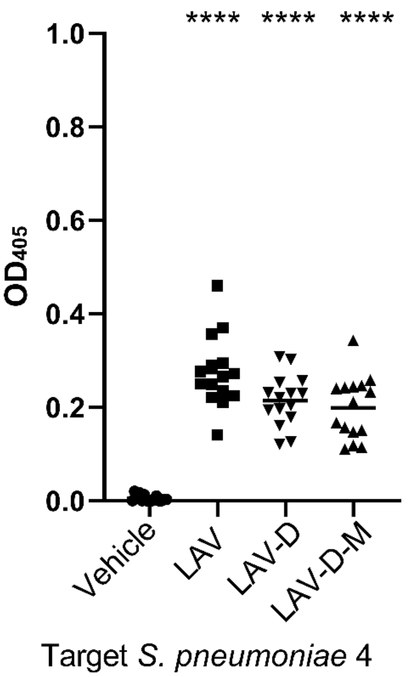

Supplemental Figure S4. Vaccination with strains expressing non-native antigenic epitopes demonstrate seroconversion against heterologous *S. pneumoniae* serotype 4. Mice were vaccinated with LAV strain, LAV-D strain, LAV-D-M strain, or PBS vehicle control, sera was collected, and IgG seroconversion in vaccinated mice was determined by ELISA against TIGR4 (serotype4). Each data point represents an individual mouse and bars represent mean. Immunoreactivity of sera of mice vaccinated with vaccine strains were compared to that of mice that received the PBS vehicle control via unpaired t-test. ****$p<0.0001$.

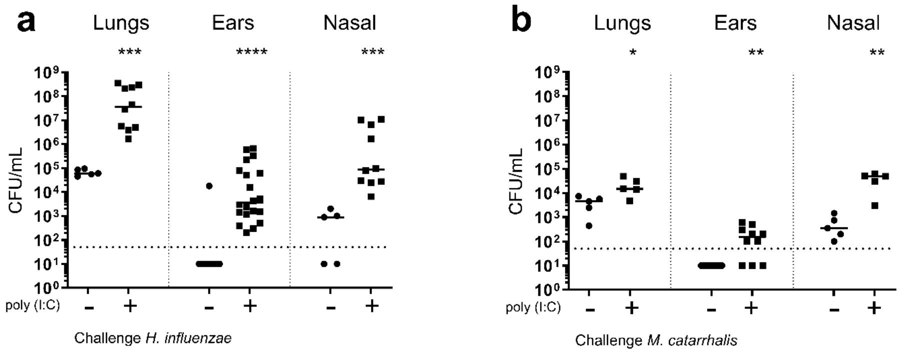

Supplemental Figure S5. Poly (I:C) treatment prior to challenge enhances localization to respiratory tissues. (**a**,**b**) Mice were treated with poly (I:C) (+) or PBS vehicle control (-) four days prior to challenge and challenged with *H. influenzae* (**a**) or *M. catarrhalis* (**b**). Bacterial burden in the lungs, ears, and nasal passage of challenged mice was determined. Each data point represents an individual mouse (lungs, nasal passage) or each ear from individual mice (ears) and bars represent median. Dashed line represents limit of detection. Bacterial burden in each tissue from mice treated with poly (I:C) was compared to that of mice that received the PBS vehicle control via non-parametric Mann-Whitney t-test. *p<0.05, **p<0.01, ***p<0.001, ****p<0.0001.

1. McCullers, J.A.; McAuley, J.L.; Browall, S.; Iverson, A.R.; Boyd, K.L.; Henriques Normark, B. Influenza enhances susceptibility to natural acquisition of and disease due to Streptococcus pneumoniae in ferrets. *J Infect Dis* **2010**, *202*, 1287-1295, doi:10.1086/656333.
2. McCullers, J.A.; Karlstrom, A.; Iverson, A.R.; Loeffler, J.M.; Fischetti, V.A. Novel strategy to prevent otitis media caused by colonizing Streptococcus pneumoniae. *PLoS Pathog* **2007**, *3*, e28, doi:10.1371/journal.ppat.0030028.
3. Iverson, A.; Meyer, C.J.; Vogel, P.; Waidyarachchi, S.; Das, N.; Bruhn, D.F.; Poole, A.; Butler, M.M.; Bowlin, T.L.; Lee, R.E.; Rosch, J.W. Efficacy of Aminomethyl Spectinomycins against Complex Upper Respiratory Tract Bacterial Infections. *Antimicrob Agents Chemother* **2019**, *63*, doi:10.1128/AAC.02096-18.
4. Echlin, H.; Rosch, J.W. Advancing Genetic Tools in Streptococcus pneumoniae. *Genes (Basel)* **2020**, *11*, doi:10.3390/genes11090965.
5. Rowe, H.M.; Karlsson, E.; Echlin, H.; Chang, T.C.; Wang, L.; van Opijnen, T.; Pounds, S.B.; Schultz-Cherry, S.; Rosch, J.W. Bacterial Factors Required for Transmission of Streptococcus pneumoniae in Mammalian Hosts. *Cell Host Microbe* **2019**, *25*, 884-891 e886, doi:10.1016/j.chom.2019.04.012.
